# Supplementary material for: Genetic testing in individuals with extreme HDL-C levels: Diagnostic yield and clinical implications from the Tromsø Study
Source: PLoS One. 2026 Apr 20;21(4):e0344627. doi: 10.1371/journal.pone.0344627 (PMC13095017; doi:10.1371/journal.pone.0344627)
Supplement: S1 File — (PDF) [file pone.0344627.s001.pdf]

S1 File. Supplementary material and methods

Plasmid construction

Construction of pcDNA3.1-WT-ABCA1-V5/his and pcDNA3.1-WT-CETP-V5 has previously been described [1, 2]. The pcDNA3.1-WT-SCARB1 and pcDNA3.1-R174C-SCARB1 constructs were a kind gift from Dr. Daisy Sahoo (Department of Medicine/Endocrinology, Medical College of Wisconsin, USA) [3]. The pCMV6-WT-LCAT-myc was from Origene Technologies (#RC203763, Rockville, MD) and the pCMV3-WT-APOA1-myc was from Sino Biological (#HG10686-CM, Bejiing, P.R. China). Genetic variants were introduced into the wild-type (WT) constructs using the QuikChange II XL Mutagenesis Kit (Agilent Technologies, Santa Clara, CA) according to the manufacturer's instructions.

Synonymous variants or variants in the non-coding regions with SpliceAI Δ-score > 0.2 were selected for functional characterization using the exon-trapping pET01 system (MoBiTec GmbH, Göttingen, Germany). Gene target regions were amplified from DNA extracted from immortalized human hepatocytes (a kind gift from Dr Philippe Costet, Paul Sabatier University, Toulouse, France) and subcloned into the cloning vector pJET1.2/blunt using the CloneJET PCR Cloning Kit (Thermo Scientific) according to the manufacturer's instructions. Utilizing single restriction sites, the pJET1.2 target regions and pET01 vector were digested, purified using Monarch® DNA Gel Extraction Kit (NEB) and ligated using the Rapid DNA Ligation Kit (Thermo Scientific) according to the manufacturer's instructions. The selected variants and controls were constructed using the Q5® Site-Directed Mutagenesis Kit (NEB) according to the manufacturer's instructions. All transgenes were verified by DNA sequencing. Oligonucleotides used for cloning and mutagenesis are available upon request.

Cell culture and transfection

HEK293 cells (European Collection of Authenticated Cell Cultures, Wiltshire, United Kingdom) and HeLa T-REx cells (Invitrogen, Carlsbad, CA) were cultured on Nunc™ Cell-Culture Treated Multidishes (Thermo Scientific) in HyClone Minimum Essential Medium (MEM; GE Healthcare Life Sciences, Pittsburg, PA) supplemented with 10% fetal bovine serum, 2 mM L-glutamine (Sigma-Aldrich), 50 U/mL penicillin, 50 µg/mL streptomycin (GE Healthcare Life Sciences) and non-essential amino acids (Biowest, Nuaillé, France). Cells were transiently transfected using FuGENE HD (Roche Diagnostics GmbH, Mannheim, Germany) in a 3:1 ratio of the amount of DNA for 24 hours (h) according to the manufacturer's instructions.

Western blot analyses

Western blot analyses on conditioned media and lysates from transiently transfected HEK293 cells were carried out as previously described [2]. In brief, cells were lysed in lysis buffer (1% Triton X-100 (Sigma-Aldrich), 150 mM NaCl and 10 mM Tris/HCl (pH 7.4)) containing Complete™ Protease Inhibitor Cocktail (Roche Diagnostics) by incubation at -80 ˚C for 30 min or sonication for ten pulses (40% amplitude, 0.5 cycle), prior to removal of cell debris by centrifugation. Conditioned medium was precleared by centrifugation prior to SDS/PAGE of equal amounts of protein was performed using 4-20% CriterionTM TGXTM Precast Gels (Bio-Rad, Hercules, CA) and blotted onto Immuno-Blot polyvinylidene difluoride membranes (Bio-Rad). The amount of ABCA1 and CETP was analyzed using a HRP conjugated anti-V5 antibody in a 1:5000 dilution (R961-25; Invitrogen). SR-BI was detected by a Rabbit Polyclonal SR-BI Antibody in a 1:5000 dilution (NB400-101SS; Bio-Techne Ltd, Abingdon, UK). The C-terminal myc-tagged LCAT was detected by a Mouse Monoclonal myc antibody in a 1:4000 dilution (#2276, Cell Signaling Technology, Danvers, MA). β-actin was used as loading control and detected by an anti-β-actin antibody from Abcam in a 1:7500 dilution (ab213262; Cambridge, UK).

Cholesterol efflux assay

The cholesterol efflux assay used to study the functional consequence of variants in ABCA1 has previously been described [1]. In brief, transiently transfected HEK293 cells were loaded with BODIPY-cholesterol (Cayman Chemicals, Ann Arbor, MI) and the ABCA1 activity was measured as level of BODIPY-cholesterol transported to crude HDL in the acceptor medium compared to level of BODIPY-cholesterol retained in the cell lysate. Cholesterol efflux from mock-transfected cells was subtracted to correct for ABCA1-nonspecific efflux.

CETP lipid transfer activity assay

Lipid transfer activity of variants in CETP was measured using the CETP Activity Assay Kit II (ab196995; Abcam, Cambridge, UK) as previously described [2].

SR-BI DiD-HDL binding and internalization assay

The method for assessing HDL-binding and -internalization capacity of SR-BI variants was adapted from May et al. [4]. In brief, HDL (ρ = 1.080–1.210 g/mL) was isolated by ultracentrifugation of serum from healthy blood donors using standard protocols and an Optima XPN-80 ultracentrifuge (Beckman Coulter Inc, Fullerton, CA). The lipid profile and protein concentration of the HDL preparation were measured at Department of Medical Biochemistry, Oslo University Hospital using standard methods. HDL was labeled with fluorescent 1,1'-Dioctadecyl-3,3,3',3'-Tetramethylindodicarbocyanine, 4-Chlorobenzenesulfonate (DiD) (Invitrogen) using the method described by Pitas et al.[5]. To assess HDL-binding, transiently transfected HEK293 cells were incubated with 1 µg/mL DiD-HDL for 1.5 h at 4°C and harvested by careful flushing in cold phosphate buffered saline with 0.5% BSA. To assess HDL-internalization, transiently transfected HEK293 cells were incubated with 5 µg/mL DiD-HDL at 37°C and harvested by trypsination to eliminate cell surface bound DiD-HDL. The amount of bound and internalized DiD-HDL was analyzed on a BD Accuri™ C6 Plus flow cytometer (BD Biosciences, San Diego, CA).

LCAT activity assay

An LCAT Activity Assay Kit (MAK107; Roar Biomedical, Bronxville, NY) was used to measure the cholesterol esterification activity of variants in LCAT according to the manufacturers' instruction. In brief, conditioned minimal medium from transiently transfected HEK293 cells was precleared, concentrated using Amicon Ultra Centrifugal Filter 30kDa (Merck, Darmstadt, Germany), and diluted 1:5 in minimal media. 3 µL of the diluted medium was used in the assay.

RT-PCR

Total RNA from HEK293 and HeLa T-Rex cells was isolated using Monarch® Total RNA Miniprep Kit (NEB). RT-PCR was performed using 0.2 µg total RNA, 0.6 µM pET01 system-suggested primers (ETPR04 5' and ETPR07 3') prolonged to adjust Tm to 60 °C annealing temperature and the Qiagen OneStep RT-PCR Kit according to the manufacturers instruction. The PCR products were analyzed by standardized DNA sequencing and gel electrophoresis using 1.5% agarose gel containing GelRed® Nucleic Acid Gel Stain (Biotium, Fremont, CA), run at 90 V for 120 min at 4 °C. All transgenes were analyzed twice in both cells types.

qPCR

Total RNA from HEK293 was isolated using Monarch® Total RNA Miniprep Kit (NEB). Isolated RNA was transcribed to cDNA, which was analyzed using PrimeTime Predesigned qPCR Assay primers (Integrated DNA Technologies, Coralville, IA); *ABCA1* (Hs.PT.58.27452429), *CETP* (Hs.PT.58.14760793), *LCAT* (Hs.PT.58.19038093), *SCARB1* (Hs.PT.58.25579929) and glyceraldehyde-3-phosphate dehydrogenase (*GAPDH*) (Hs.PT.39a.22214836). mRNA amounts were determined and normalized to the housekeeping gene *GAPDH* by the $2^{-\Delta\Delta Ct}$ method [6].

Distribution of HDL-C levels and number of individuals in the extreme of the spectrum

Sex specific HDL-C distribution data and the number of individuals with HDL-C levels above or below specific thresholds were derived from laboratory registry data from Fürst Medical Laboratory, Oslo, the largest medical laboratory in Norway. The HDL-C measurements pertain to individuals aged 18–49.9 years, primarily requisitioned by general practitioners, and thus, the sample is not necessarily representative of the general population (85% South-East Norway). All blood samples were analyzed at the central laboratory in Oslo, Norway. Approximately 10% of individuals had two HDL-C measurements in 2019, for whom the values are reported as the average for that year. Details on the laboratory's methodology and data collection procedures have previously been described [7].

References

1. Teigen M, Ølnes Å S, Bjune K, Leren TP, Bogsrud MP, Strøm TB. Functional characterization of missense variants affecting the extracellular domains of ABCA1 using a fluorescence-based assay. J Lipid Res. 2024;65(1):100482.

2. Ølnes Å S, Teigen M, Laerdahl JK, Leren TP, Strøm TB, Bjune K. Variants in the CETP gene affect levels of HDL cholesterol by reducing the amount, and not the specific lipid transfer activity, of secreted CETP. PLoS One. 2023;18(12):e0294764.

3. Chadwick AC, Sahoo D. Functional characterization of newly-discovered mutations in human SR-BI. PLoS One. 2012;7(9):e45660.

4. May SC, Dron JS, Hegele RA, Sahoo D. Human variant of scavenger receptor BI (R174C) exhibits impaired cholesterol transport functions. J Lipid Res. 2021;62:100045.

5. Pitas RE, Innerarity TL, Weinstein JN, Mahley RW. Acetoacetylated lipoproteins used to distinguish fibroblasts from macrophages in vitro by fluorescence microscopy. Arteriosclerosis. 1981;1(3):177–85.

6. Livak KJ, Schmittgen TD. Analysis of relative gene expression data using real-time quantitative PCR and the 2(-Delta Delta C(T)) Method. Methods. 2001;25(4):402–8.

7. Arnesen EK, Retterstol K. Secular trends in serum lipid profiles in young adults in Norway, 2001-19. Atheroscler Plus. 2022;48:60–7.
