# Supplementary material for: Genetic testing in individuals with extreme HDL-C levels: Diagnostic yield and clinical implications from the Tromsø Study
Source: PLoS One. 2026 Apr 20;21(4):e0344627. doi: 10.1371/journal.pone.0344627 (PMC13095017; doi:10.1371/journal.pone.0344627)
Supplement: S1 Table — All individuals are indicated with sex, age group at first entry, lipid values (mmol/L) at the Tromsø 4 (1994–1995), 5 (2001), 6 (2007–2008) and 7 (2015–2016) studies and the mean HDL-cholesterol values. (PDF) [file pone.0344627.s002.pdf]

S1 Table. An overview of all the individuals included in this study.

			Total cholesterol				HDL-cholesterol				
**Individual**	**Sex**	**Age group**	**Tromsø 4**	**Tromsø 5**	**Tromsø 6**	**Tromsø 7**	**Tromsø 4**	**Tromsø 5**	**Tromsø 6**	**Tromsø 7**	**Mean**
**Low HDL-cholesterol**											
1	Male	50 - 59	6.23				0.36				0.36
2	Male	40 - 49				3.20				0.40	0.40
3	Male	40 - 49	2.72				0.46				0.46
4	Male	30 - 39	4.74			5.60	0.54			0.40	0.47
5	Male	50 - 59	6.26	3.44	2.70	2.30	0.69	0.53	0.50	0.40	0.53
6	Male	50 - 59	4.10	4.10			0.63	0.47			0.55
7	Male	40 - 49	5.16	6.43			0.49	0.63			0.56
8	Male	40 - 49	3.97		6.50		0.78		0.40		0.59
9	Male	40 - 49	4.31	4.59	3.40	2.20	0.76	0.59	0.60	0.46	0.60
10	Male	20 - 29	4.93		5.60	3.40	0.48		0.70	0.68	0.62
11	Male	50 - 59	7.47	6.50	5.50	3.60	0.87	0.73	0.70	0.43	0.68
12	Female	40 - 49	6.58		7.00	4.60	1.36		1.40	0.20	0.99
**High HDL-cholesterol**											
13	Male	50 - 59	7.84		4.50	4.80	1.46		1.40	3.60	2.15
14	Male	50 - 59	7.54		3.70	4.60	1.30		1.60	3.60	2.17
15	Female	20 - 29	5.18	4.73	5.20	7.00	1.66	1.77	2.20	3.30	2.23
16	Female	50 - 59	3.98	4.53	4.90	5.00	1.58	2.17	2.10	3.10	2.24
17	Female	40 - 49	6.51			6.80	1.38			3.10	2.24
18	Female	50 - 59	6.39	4.65	4.60	4.50	1.91	1.80	2.10	3.30	2.28
19	Female	40 - 49	5.02		8.10	4.90	1.75		2.10	3.03	2.29
20	Female	40 - 49		4.94		4.90		1.50		3.10	2.30
21	Male	60 - 69	6.70	7.91			1.58	3.05			2.32
22	Female	50 - 59	7.41	5.60	5.40	7.80	1.76	1.88	2.00	3.70	2.34
23	Female	70 - 79	6.02	7.31			3.20	1.52			2.36
24	Female	60 - 69	7.44	6.77	8.80		1.81	1.85	3.60		2.42
25	Female	50 - 59	7.15	8.24	8.90		2.13	1.86	3.30		2.43
26	Female	50 - 59	5.65	5.64	7.30	6.00	2.04	2.00	3.10	2.60	2.44
27	Female	30 - 39	6.06		6.90	7.20	1.74		2.00	3.60	2.45
28	Male	50 - 59	8.18	6.86	5.80	6.40	1.73	1.97	3.30	2.80	2.45
29	Female	30 - 39	5.03	5.09		6.20	2.22	2.05		3.10	2.46

Continued

S1 Table *Continued*

Individual	Sex	Age group	Total cholesterol				HDL-cholesterol				
			Tromsø 4	Tromsø 5	Tromsø 6	Tromsø 7	Tromsø 4	Tromsø 5	Tromsø 6	Tromsø 7	Mean
30	Female	40 - 49	5.08		6.20	6.60	1.97		2.40	3.10	2.49
31	Female	60 - 69	7.57	10.03	8.60	7.60	1.92	3.06	2.80	2.20	2.50
32	Female	30 - 39	5.55			7.90	1.91			3.09	2.50
33	Female	50 - 59	6.27	6.57	4.40	6.50	2.34	2.26	1.90	3.50	2.50
34	Female	30 - 39	5.22			6.90	1.81			3.20	2.51
35	Female	50 - 59	4.81	5.58	6.10	6.90	2.24	1.98	2.80	3.03	2.51
36	Male	50 - 59	5.15			6.60	1.74			3.30	2.52
37	Female	40 - 49	5.89	6.44	5.70		3.04	2.53	2.00		2.52
38	Female	30 - 39	5.43			8.80	1.95			3.10	2.53
39	Male	50 - 59	5.92	6.25	4.60	3.60	2.68	2.42	3.30	1.70	2.53
40	Female	50 - 59	7.90	7.55	7.40	4.90	2.27	2.44	2.30	3.10	2.53
41	Female	20 - 29	5.15			5.50	1.96			3.10	2.53
42	Female	40 - 49	6.77		8.10	5.20	2.10		2.40	3.10	2.53
43	Female	60 - 69	5.12		6.40		1.68		3.40		2.54
44	Female	40 - 49	5.76		5.90	6.50	2.02		2.20	3.40	2.54
45	Female	20 - 29	4.18			5.40	1.99			3.10	2.55
46	Female	40 - 49	4.96		5.90	6.70	1.71		2.30	3.63	2.55
47	Female	50 - 59	7.64	8.82	6.20		2.11	2.33	3.20		2.55
48	Female	50 - 59	7.15	5.91	5.10		3.40	1.94	2.30		2.55
49	Male	30 - 39	6.12		5.40	5.50	1.74		2.50	3.40	2.55
50	Male	50 - 59	6.51	5.26	5.10		3.06	2.49	2.10		2.55
51	Female	40 - 49	5.26		6.40	6.20	2.07		2.50	3.10	2.56
52	Female	50 - 59	7.08	7.42	6.00		3.20	2.43	2.10		2.58
53	Female	40 - 49	5.86		5.50	7.30	2.24		2.20	3.30	2.58
54	Male	60 - 69	7.23	6.56			3.04	2.17			2.61
55	Female	50 - 59	7.46	6.58	6.90	4.70	2.50	2.06	2.70	3.20	2.62
56	Female	70 - 79	5.78	3.98			3.26	1.99			2.63
57	Female	40 - 49	5.24			6.20	1.94			3.38	2.66
58	Female	60 - 69	7.97	7.47			3.02	2.30			2.66
59	Female	40 - 49	6.20			8.40	2.22			3.10	2.66
60	Female	40 - 49	5.71		6.40	7.40	2.20		2.20	3.58	2.66
61	Female	30 - 39	4.77	4.60	4.90	5.10	2.38	2.22	2.70	3.40	2.68
62	Male	20 - 29	3.66		4.50	5.10	2.14		2.50	3.40	2.68
63	Female	60 - 69	8.98	6.51	9.20		2.46	2.01	3.60		2.69
64	Female	50 - 59	5.76	6.59	7.30	5.20	2.09	2.69	2.90	3.08	2.69
65	Female	50 - 59	5.79	6.08	5.60	6.70	2.45	2.38	2.40	3.55	2.70

Continued

S1 Table *Continued*

			Total cholesterol				HDL-cholesterol				
**Individual**	**Sex**	**Age group**	**Tromsø 4**	**Tromsø 5**	**Tromsø 6**	**Tromsø 7**	**Tromsø 4**	**Tromsø 5**	**Tromsø 6**	**Tromsø 7**	**Mean**
66	Female	40 - 49	6.58			7.20	2.10			3.30	2.70
67	Female	40 - 49	6.17		6.00	5.60	2.01		2.70	3.40	2.70
68	Female	50 - 59	6.88	7.02	6.70	7.30	2.33	2.66	2.20	3.69	2.72
69	Female	40 - 49	4.34		4.90	4.80	2.57		2.50	3.10	2.72
70	Female	40 - 49	10.30		5.70	6.90	2.29		2.40	3.50	2.73
71	Female	50 - 59	7.25	6.75			3.08	2.38			2.73
72	Male	60 - 69	6.79	6.99	6.90		2.06	2.59	3.60		2.75
73	Male	60 - 69	7.88	7.19			3.08	2.43			2.76
74	Female	40 - 49	5.34		5.30	5.70	2.37		2.70	3.20	2.76
75	Female	40 - 49	4.79			7.20	2.42			3.10	2.76
76	Male	40 - 49	6.01			6.70	2.02			3.50	2.76
77	Female	60 - 69	5.10	5.36			2.47	3.05			2.76
78	Female	40 - 49	4.75		6.10	6.10	2.09		2.90	3.30	2.76
79	Female	40 - 49	5.59			6.00	3.04			2.50	2.77
80	Female	30 - 39	5.16			5.70	2.14			3.40	2.77
81	Female	40 - 49		4.75		5.70		2.34		3.20	2.77
82	Female	40 - 49	5.64		6.40	6.70	2.44		2.60	3.30	2.78
83	Female	50 - 59	4.56		5.70		2.46		3.10		2.78
84	Female	50 - 59	6.29	5.65	4.60	5.10	2.79	2.66	2.70	3.02	2.79
85	Female	50 - 59	5.30	5.56	5.50	6.10	2.79	2.69	2.60	3.10	2.80
86	Female	20 - 29	7.01		4.70	6.50	2.80		2.20	3.40	2.80
87	Female	60 - 69	7.07	6.77	6.90		2.50	2.41	3.50		2.80
88	Female	50 - 59	6.57	6.16	5.50	6.20	2.22	2.26	3.00	3.80	2.82
89	Male	50 - 59	5.65	6.08	5.70	5.50	2.55	2.54	2.80	3.40	2.82
90	Female	30 - 39	5.42			7.20	2.45			3.20	2.83
91	Female	40 - 49	6.79			7.00	2.06			3.60	2.83
92	Female	40 - 49	9.03		7.80	8.30	3.02		2.50	3.00	2.84
93	Female	30 - 39	4.50			5.70	2.29			3.40	2.85
94	Female	60 - 69			6.00	6.40			2.60	3.10	2.85
95	Female	30 - 39	6.29		6.20	8.20	2.36		2.90	3.30	2.85
96	Female	40 - 49	5.77			7.90	2.31			3.40	2.86
97	Male	50 - 59	6.24		6.00	6.80	2.50		2.50	3.58	2.86
98	Female	30 - 39	6.48	6.77	7.70		2.80	2.43	3.40		2.88
99	Female	50 - 59	5.80	6.21	4.90	5.90	2.70	3.01	2.30	3.50	2.88
100	Female	30 - 39	6.38			6.30	2.66			3.10	2.88
101	Male	60 - 69	5.81	7.02			2.73	3.03			2.88
102	Female	30 - 39	5.65		6.30	6.10	2.04		3.20	3.40	2.88

Continued

S1 Table *Continued*

Individual	Sex	Age group	Total cholesterol				HDL-cholesterol				
			Tromsø 4	Tromsø 5	Tromsø 6	Tromsø 7	Tromsø 4	Tromsø 5	Tromsø 6	Tromsø 7	Mean
103	Female	50 - 59	7.09	8.92	7.40	6.50	2.72	2.91	2.50	3.40	2.88
104	Male	50 - 59	6.45		6.00		2.67		3.10		2.89
105	Female	30 - 39	4.75	5.36	5.70	6.40	2.29	2.56	2.90	3.80	2.89
106	Male	60 - 69			6.40	7.20			2.70	3.10	2.90
107	Female	50 - 59	5.07	6.16	6.40	6.70	2.50	2.82	3.30	3.00	2.91
108	Female	40 - 49	5.52			5.80	2.22			3.60	2.91
109	Female	30 - 39	6.33			6.20	2.72			3.10	2.91
110	Male	60 - 69	6.32	7.31	7.70		2.77	2.66	3.30		2.91
111	Female	40 - 49	6.98			7.30	2.74			3.10	2.92
112	Female	40 - 49	6.85			7.10	2.16			3.70	2.93
113	Female	50 - 59	7.35	7.08			2.78	3.09			2.94
114	Female	60 - 69	6.29	5.72	5.60	5.30	2.96	2.72	3.20	2.90	2.95
115	Male	40 - 49	6.37		6.00		2.79		3.10		2.95
116	Female	40 - 49	5.43		5.10	6.40	2.91		2.60	3.33	2.95
117	Female	30 - 39	6.86		6.50	7.60	2.75		2.70	3.40	2.95
118	Male	50 - 59	6.86		6.90	7.90	2.83		2.80	3.30	2.98
119	Female	50 - 59	7.40	7.20	6.40	5.90	3.10	3.01	3.00	2.80	2.98
120	Female	40 - 49	8.36		8.70	5.90	2.66		3.50	2.80	2.99
121	Female	50 - 59	5.25	6.28	5.50	5.40	2.63	2.97	2.80	3.60	3.00
122	Female	60 - 69			5.60	7.20			2.70	3.30	3.00
123	Female	40 - 49	5.03			6.00	2.40			3.60	3.00
124	Female	60 - 69	9.68	6.84	5.90	7.90	2.78	2.44	2.60	4.20	3.01
125	Female	50 - 59	7.38	6.99	6.60	7.00	2.85	2.60	3.10	3.50	3.01
126	Male	40 - 49	6.25			6.00	2.15			3.90	3.03
127	Female	30 - 39	5.12	5.04		5.10	2.85	2.73		3.50	3.03
128	Female	40 - 49				6.90				3.03	3.03
129	Female	60 - 69	8.45	7.87			3.04	3.02			3.03
130	Female	60 - 69	8.72	9.20			2.97	3.10			3.04
131	Female	40 - 49	8.33				3.04				3.04
132	Female	40 - 49	5.33			6.40	2.99			3.10	3.05
133	Male	40 - 49	6.90			5.80	2.80			3.30	3.05
134	Female	70 - 79	6.49	6.72		6.40	3.00	2.25		3.90	3.05
135	Male	50 - 59	6.63				3.06				3.06
136	Female	50 - 59	7.04				3.06				3.06
137	Male	40 - 49				5.70				3.06	3.06
138	Female	30 - 39	4.93		6.50	5.40	2.19		4.00	3.00	3.06
139	Female	50 - 59	9.13	8.13			3.24	2.92			3.08

Continued

S1 Table *Continued*

			Total cholesterol				HDL-cholesterol				
**Individual**	**Sex**	**Age group**	**Tromsø 4**	**Tromsø 5**	**Tromsø 6**	**Tromsø 7**	**Tromsø 4**	**Tromsø 5**	**Tromsø 6**	**Tromsø 7**	**Mean**
140	Female	80 - 89	6.18				3.08				3.08
141	Female	50 - 59	5.81		6.10		3.16		3.00		3.08
142	Female	40 - 49	4.78		5.80	6.80	2.35		3.10	3.80	3.08
143	Male	50 - 59	7.88		7.00	6.60	2.78		2.30	4.20	3.09
144	Female	30 - 39			5.40				3.10		3.10
145	Female	40 - 49				5.60				3.10	3.10
146	Female	60 - 69				7.00				3.10	3.10
147	Female	50 - 59				7.50				3.10	3.10
148	Male	60 - 69				4.30				3.10	3.10
149	Female	50 - 59				6.00				3.10	3.10
150	Female	80 - 89	6.86				3.10				3.10
151	Female	70 - 79				5.90				3.12	3.12
152	Female	30 - 39	7.13				3.12				3.12
153	Female	50 - 59	6.52	5.90	6.80	6.10	3.40	1.79	2.90	4.40	3.12
154	Female	40 - 49	5.09			5.70	2.86			3.40	3.13
155	Female	40 - 49	6.14		5.40	6.20	2.93		2.90	3.59	3.14
156	Female	40 - 49	7.73			7.30	3.28			3.00	3.14
157	Female	80 - 89	8.62				3.14				3.14
158	Female	50 - 59			5.40	5.60			2.90	3.40	3.15
159	Female	40 - 49	9.39			6.80	2.80			3.50	3.15
160	Female	30 - 39	5.08			6.40	2.14			4.20	3.17
161	Female	60 - 69	5.79	6.81	6.50		2.79	3.34	3.40		3.18
162	Female	30 - 39	6.41	6.01		5.30	3.12	3.42		3.00	3.18
163	Female	50 - 59				8.90				3.20	3.20
164	Female	40 - 49				5.90				3.20	3.20
165	Male	60 - 69			6.20	6.30			1.80	4.60	3.20
166	Female	60 - 69			5.50	7.60			2.80	3.60	3.20
167	Female	50 - 59			6.20				3.20		3.20
168	Female	60 - 69	6.49				3.20				3.20
169	Female	50 - 59				5.60				3.20	3.20
170	Female	50 - 59				7.80				3.20	3.20
171	Female	50 - 59	6.04	7.75	6.80	7.40	2.59	2.93	3.00	4.30	3.21
172	Female	50 - 59	7.05	6.71	6.70		2.76	2.78	4.10		3.21
173	Female	40 - 49	6.18		6.30	8.50	2.78		2.70	4.20	3.23
174	Female	40 - 49	5.61			7.20	2.87			3.60	3.24
175	Female	60 - 69	8.54	9.28			3.28	3.19			3.24
176	Male	50 - 59	7.40	8.94	8.10		2.39	2.72	4.60		3.24

Continued

S1 Table *Continued*

Individual	Sex	Age group	Total cholesterol				HDL-cholesterol				
			Tromsø 4	Tromsø 5	Tromsø 6	Tromsø 7	Tromsø 4	Tromsø 5	Tromsø 6	Tromsø 7	Mean
177	Male	50 - 59	7.17		7.20		2.92		3.60		3.26
178	Female	40 - 49	5.54		6.40	6.50	2.94		3.50	3.40	3.28
179	Female	50 - 59				7.90				3.28	3.28
180	Female	70 - 79	7.37				3.28				3.28
181	Female	50 - 59	5.57	6.57	6.50	6.30	2.55	2.85	3.90	3.90	3.30
182	Female	80 - 89	9.24				3.30				3.30
183	Female	40 - 49				5.90				3.30	3.30
184	Male	50 - 59	7.25	6.88	5.90	6.10	2.67	3.25	3.70	3.70	3.33
185	Female	40 - 49	4.63		5.90	6.50	2.70		3.30	4.00	3.33
186	Male	40 - 49	7.95				3.38				3.38
187	Male	40 - 49	5.95				3.38				3.38
188	Female	40 - 49	9.10		10.60	11.20	2.34		3.80	4.00	3.38
189	Female	40 - 49	5.36		7.60		2.47		4.30		3.39
190	Female	40 - 49	5.98			6.90	2.99			3.80	3.40
191	Female	40 - 49				5.90				3.40	3.40
192	Female	40 - 49				6.20				3.40	3.40
193	Female	60 - 69				9.00				3.40	3.40
194	Female	40 - 49				5.60				3.40	3.40
195	Female	70 - 79			6.10				3.40		3.40
196	Female	50 - 59			5.90	6.20			3.30	3.60	3.45
197	Female	60 - 69	5.97	6.60	5.90	6.50	2.97	3.29	3.50	4.10	3.47
198	Male	40 - 49				6.70				3.50	3.50
199	Female	60 - 69				6.50				3.50	3.50
200	Female	50 - 59				5.50				3.60	3.60
201	Female	40 - 49	5.40			7.70	2.69			4.80	3.75
202	Female	50 - 59				6.70				3.80	3.80
203	Female	40 - 49	7.15			7.80	2.52			5.20	3.86
204	Female	40 - 49				5.30				3.90	3.90
205	Male	60 - 69	5.55				3.90				3.90
206	Female	40 - 49	8.76			8.50	3.40			4.50	3.95
207	Female	40 - 49	5.46			7.20	2.49			5.60	4.05
208	Female	40 - 49	6.30			8.10	2.94			5.20	4.07
209	Female	40 - 49			6.80				4.40		4.40
210	Female	50 - 59				6.80				4.90	4.90
