# Supplementary material for: Genetic testing in individuals with extreme HDL-C levels: Diagnostic yield and clinical implications from the Tromsø Study
Source: PLoS One. 2026 Apr 20;21(4):e0344627. doi: 10.1371/journal.pone.0344627 (PMC13095017; doi:10.1371/journal.pone.0344627)
Supplement: S2 Table — Missense variants in ABCA1 (NM_005502.4), APOA1 (NM_000039.3), CETP (NM_000078.3), LCAT (NM_000229.2), PLTP (NM_006227.4) and SCARB1 (NM_005505.5) are annotated with respect to their effects at protein and nucleotide level. The reported lipid associated phenotypic effects of the variants, as well as the classifications made by Human Gene Mutation Database (HGMD) for variants reported in that database are shown. Also shown is an in silico prediction of pathogenicity represented by a REVEL score [66]. Allele frequencies of the variants are obtained from the Genome Aggregation Database (gnomAD, v.4.1.0). Pathogenicity classes of the variants were assessed according to the guidelines from The American College of Medical Genetics and Genomics and The Association for Molecular Pathology (ACMG), and the criteria used are indicated [21]. The number and percentage of variant carriers among the Tromsø study participants with high or low HDL-C are also indicated. HDL-C: HDL-cholesterol level. aHGMD HDL-related phenotype associated with the individual variant. bHGMD class: DM: Disease-causing mutation; DM?: Disease-causing mutation?; DP: Disease-associated polymorphism; FP: in vivo or in vitro functional polymorphism; DFP: Disease-associated polymorphism with supporting functional evidence; -: Not listed in HGMD. cA higher REVEL score (from 0 to 1) indicates a greater likelihood of a deleterious variant. dThe highest allele frequency among the populations: African/African American (AFR), Amish (AMI), Admixed American (AMR), Ashkenazi Jewish (ASJ), East Asian (EAS), Finnish (FIN), Middle Eastern (MID), Non-Finnish European (NFE) and South Asian (SAS) is shown. eClass 1: benign; Class 2: likely benign; Class 3: Unknown significance; Class 4: likely pathogenic; Class 5: pathogenic. fCriteria for pathogenicity weighed as strong (PS4, PM3_str, PP4_str), moderate (PS3_mod, PM2) or supporting (PS3–4_sup, PP3) and criteria for benignity weighed as stand-alone (BA1), strong (BS1–2), mod [file pone.0344627.s003.pdf]

S2 Table. All missense variants in HDL-related genes in participants from the Tromsø Study.

Variant		HGDM				ACMG		Phenotype Tromsø [HDL-C] n (%)	
**Protein**	**Nucleotide**	**HDL-C[a]**	**Class[b]**	**REVEL score[c]**	**gnomAD[d]**	**Class[e]**	**Criteria[f]**	**High**	**Low**
**ABCA1**									
p.R219K	c.656G>A	High	FP	0.187	1/2 (AFR)	1	BA1, BS3_mod, BP4	86 (43.4%)	6 (50.0%)
p.S296T	c.886T>A	-	-	0.387	1/1602 (EAS)	2	BS3_mod, BP4	1 (0.5%)	-
p.V771M	c.2311G>A	-	FP	0.181	1/6 (AFR)	1	BA1, BS3_mod, BP4	11 (5.6%)	2 (16.7%)
p.V825I	c.2473G>A	High	FP	0.195	1/3 (EAS)	1	BA1, BS3_mod, BP4	25 (12.6%)	2 (16.7%)
p.I883M	c.2649A>G	-	-	0.156	1/2 (EAS)	1	BA1, BS3_mod, BP4	37 (18.7%)	2 (16.7%)
p.C887F	c.2660G>T	High	DM	0.488	1/143 (EAS)	1	BA1, BS3_mod	2 (1.0%)	-
p.E1172D	c.3516G>C	-	DP	0.332	1/6 (AFR)	1	BA1, BP4	12 (6.1%)	1 (8.30%)
p.S1181F	c.3542C>T	Low	DM?	0.605	1/263 (FIN)	3	-	1 (0.5%)	-
p.K1587R	c.4760A>G	-	FP	0.360	1/1 (FIN)	1	BA1, BP4	132 (66.7%)	4 (33.3%)
p.V1674I	c.5020G>A	Low	DM	0.457	1/128 (EAS)	1	BA1	2 (1.0%)	-
p.G1818E	c.5453G>A	-	-	0.958	1/393 303 (NFE)	4	PS3_mod, PS4_sup, PM2, PP3	-	2 (16.7%)
p.R1925Q	c.5774G>A	Low	DM	0.436	1/94 (FIN)	2	BS2, BS3_mod, BP4	4 (2.0%)	-
**APOA1**									
p.E100Q	c.298G>C	-	-	0.373	1/295 009 (NFE)	3	PM2, BP4	1 (0.5%)	-
p.R184L	c.551G>T	Low	DM	0.806	1/589 187 (NFE)	4	PS3_sup, PS4, PM2, PP3	-	1 (8.30%)
p.A188S	c.562G>T	-	DP	0.104	1/973 (NFE)	3	BP4	2 (1.0%)	-
**CETP**									
p.A15G	c.44C>G	-	DP	0.013	1/15 (AFR)	1	BA1, BS3_mod, BP4	3 (1.5%)	-
p.D131N	c.391G>A	High	DM?	0.188	1/14 568 (NFE)	3	PM2, BS3_mod, BP4	1 (0.5%)	-
p.L290P	c.869T>C	-	DP	0.326	1/4322 (NFE)	3	PS3_mod	1 (0.5%)	-
p.Q337*	c.1009C>T	-	-	-	1/91 088 (SAS)	5	PVS1, PS3_mod, PS4, PM2	4 (2.0%)	-
p.V385M	c.1153G>A	-	-	0.032	1/191 (AFR)	2	BS1, BS3_mod, BP4	1 (0.5%)	-
p.A390P	c.1168G>C	Low	FP	0.136	1/10 (AMR)	1	BA1	9 (4.5%)	1 (8.30%)
p.V422I	c.1264G>A	High	DFP	0.013	1/1 (NFE)	1	BA1, BP4	171 (86.4%)	8 (66.7%)

p.E443K	c.1327G>A	High	FP	0.195	1/15 007 (AMR)	3	PS3_mod, PM2	1 (0.5%)	-
p.D459G	c.1376A>G	High	DM	0.134	1/30 (EAS)	5	PS3_mod, PS4, PP4_str	1 (0.5%)	-
p.R468Q	c.1403G>A	High	FP	0.012	1/11 (AMR)	1	BA1, BS3_mod, BP4	3 (1.5%)	1 (8.30%)
**LCAT**									
p.S232T	c.694T>A	Low	DP	0.571	1/31 (NFE)	1	BA1	7 (3.5%)	3 (25.0%)
p.M276K	c.827T>A	Low	DM	0.933	1/1 180 014 (NFE)	5	PS3_sup, PM2, PM3_str, PP3, PP4_str	-	2 (16.7%)
p.E378K	c.1132G>A	-	-	0.375	1/92 (FIN)	3	BP4	1 (0.5%)	-
**SCARB1**									
p.G2S	c.4G>A	-	DFP	0.178	1/6 (ASJ)	1	BA1, BS3_mod, BP4	28 (14.1%)	1 (8.30%)
p.G12R	c.34G>A	-	-	0.053	1/287 592 (NFE)	3	PM2, BS3_mod, BP4	1 (0.5%)	-
p.V135I	c.403G>A	-	DP	0.079	1/24 (SAS)	1	BA1, BP4	1 (0.5%)	-
p.I231V	c.691A>G	-	-	0.069	-	3	PM2, BS3_mod, BP4	1 (0.5%)	-
**PLTP**									
p.E211Q	c.631G>C	-	-	0.033	1/6591 (NFE)	3	PM2, BP4	3 (1.5%)	-
p.R380W	c.1138C>T	-	DM?	0.129	1/64 (ASJ)	3	BP4	2 (1%)	-
p.V422M	c.1264G>A	-	-	0.216	1/100 (ASJ)	3	BP4	2 (1%)	-
