# Supplementary material for: Genetic testing in individuals with extreme HDL-C levels: Diagnostic yield and clinical implications from the Tromsø Study
Source: PLoS One. 2026 Apr 20;21(4):e0344627. doi: 10.1371/journal.pone.0344627 (PMC13095017; doi:10.1371/journal.pone.0344627)
Supplement: S3 Table — Variants are annotated with respect to their effects at the protein and nucleotide level. The reported lipid associated phenotypic effects of the variants, as well as the classifications made by the Human Gene Mutation Database (HGMD) are shown. Also indicated are allele frequencies of the variants obtained from the Genome Aggregation Database (gnomAD, v4.1.0). Pathogenicity classes of the variants were assessed according to the guidelines from The American College of Medical Genetics and Genomics and The Association for Molecular Pathology (ACMG), and the criteria used are indicated [21]. The number and percentage of variant carriers among the Tromsø Study participants with high or low HDL-C are also indicated. HDL-C: HDL-cholesterol level. aHGMD reported phenotype associated with the individual variant. bHGMD class: DM: Disease-causing mutation; DM?: Disease-causing mutation?; DP: Disease-associated polymorphism; FP: in vivo or in vitro functional polymorphism; DFP: Disease-associated polymorphism with supporting functional evidence; -: Not listed in HGMD. cThe highest allele frequency among the populations: African/African American (AFR), Amish (AMI), Admixed American (AMR), Ashkenazi Jewish (ASJ), East Asian (EAS), Finnish (FIN), Middle Eastern (MID), Non-Finnish European (NFE) and South Asian (SAS) is shown. dClass 1: benign; Class 2: likely benign; Class 3: Unknown significance; Class 4: likely pathogenic; Class 5: pathogenic. eCriteria for pathogenicity weighed as strong (PS4, PM3_str, PP4_str), moderate (PS3_mod, PM2) or supporting (PS3_sup, PP3–4) and criteria for benignity weighed as stand alone (BA1), strong (BS1–2), moderate (BS3_mod) or supporting (BP4, BP7). fSpliceAI reports Δ scores ranging from 0 to 1 reporting the probability of splice alteration induced by the variant [67]. (PDF) [file pone.0344627.s004.pdf]

S3 Table. All silent or intronic variants in HDL-related genes in participants from the Tromsø Study.

Variant		HGMD			ACMG		Phenotype Tromsø [HDL-C] n (%)	
Protein	Nucleotide	HDL-C[a]	Class[b]	GnomAD[c]	Class[d]	Criteria[e]	High	Low
**Variants with SpliceAI Δ score[f] > 0.2**								
**CETP**								
-	c.1321+1G>A	-	DM	1/371 (EAS)	5	PVS1, PS4, PP4_str	1 (0.5%)	-
**SCARB1**								
p.G197G	c.591C>T	-	-	-	3	PM2, PS3_sup	1 (0.5%)	-
**Variants with SpliceAI Δ score[f] < 0.2**								
**ABCA1**								
-	c.-18G>C	-	-	1/4 (SAS)	1	BA1, BP4	32 (16.2%)	4 (33.3%)
p.L33L	c.99A>G	-	-	1/314 (FIN)	2	BP4, BP7	1 (0.5%)	-
p.L158L	c.474G>A	Low	DM?	1/2 (EAS)	1	BA1, BP4, BP7	84 (42.4%)	5 (41.7%)
-	c.720+6T>C	Low	DM	1/618 (NFE)	3	-	-	1 (8.3%)
-	c.814-14dupA	-	-	2/3 (EAS)	1	BA1, BS3	28 (14.1%)	2 (16.7%)
p.P312P	c.936C>T	-	-	1/5 (AMI)	1	BA1, BP4, BP7	30 (15.2%)	2 (16.7%)
p.G316G	c.948G>A	-	-	1/4 (AFR)	1	BA1, BP4, BP7	47 (23.7%)	4 (33.3%)
-	c.1195-8G>A	-	-	1/542 (FIN)	3	BS2	6 (3.0%)	-
-	c.1195-13C>T	-	-	1/3 (EAS)	1	BA1	27 (13.6%)	2 (16.7%)
p.T459T	c.1377A>C	-	-	1/272 (EAS)	2	BS1, BP4, BP7	2 (1.0%)	-
p.Y627Y	c.1881C>T	-	-	1/1419 (FIN)	2	BS2, BP4, BP7	7 (3.5%)	-
p.I680I	c.2040C>A	-	-	1/2 (EAS)	1	BA1, BP4, BP7	38 (19.2%)	2 (16.7%)
p.S723S	c.2169C>T	-	-	1/147 (FIN)	3	-	1 (0.5%)	-
-	c.2338-17T>C	-	-	1/67 (SAS)	1	BA1	1 (0.5%)	-
-	c.2960+18G>A	-	-	1/12 (AFR)	1	BA1, BP4	1 (0.5%)	-
p.V1053V	c.3159T>G	-	-	1/84 (NFE)	1	BA1, BP4, BP7	3 (1.5%)	-
p.E1211E	c.3633A>G	-	-	1/9 (AFR)	1	BA1, BP4, BP7	1 (0.5%)	-
p.T1427T	c.4281G>A	-	-	1/3 (EAS)	1	BA1, BP4, BP7	33 (16.7%)	1 (8.3%)
p.T1512T	c.4536G>T	-	-	1/32 (FIN)	1	BA1	8 (4.0%)	-
-	c.4773+12T>C	-	-	1/30984 (NFE)	3	PM2, BP4	1 (0.5%)	-
-	c.5927+18T>C	-	-	1/2 (AFR)	1	BA1, BP4	38 (19.2%)	3 (25.0%)
-	c.6070-63_6070-62insATGTGTAGGACATGT	-	-	1/29 (AMI)	2	BS1, BP4	1 (0.5%)	-
p.G2061G	c.6183C>T	-	-	1/4 (AFR)	1	BA1, BP4, BP7	3 (1.5%)	-
-	c.6205-39delT	Low	DM	1/228 (AMI)	3	PP4, BP4	1 (0.5%)	-
-	c.6401+13A>G	-	-	1/3 (EAS)	1	BA1, BP4	16 (8.1%)	1 (8.3%)

**APOA1**								
-	c.-12C>T	-	-	1/44215 (NFE)	3	PM2, BP4	-	1 (8.3%)
**CETP**								
-	c.658+8C>T	-	-	1/2 (AMI)	1	BA1, BP4	159 (80.3%)	9 (75.0%)
-	c.1215-14C>T	-	-	1/3 (NFE)	1	BA1, BP4	116 (58.6%)	5 (41.7%)
p.S268S	c.804G>T	-	-	1/682 (NFE)	2	BP4, BP7	2 (1.0%)	-
p.F287F	c.861C>T	-	-	1/8 (ASJ)	1	BA1	33 (17.6%)	-
p.T339T	c.1017C>T	-	-	1/4155 (NFE)	2	BP4, BP7	2 (1.0%)	1 (8.3%)
p.T387T	c.1161C>T	-	-	1/144 (AFR)	1	BA1, BP4, BP7	2 (1.0%)	-
-	c.659-109delA	-	-	1/64 (ASJ)	2	BS1, BP4	2 (1.0%)	-
**LCAT**								
p.L393L	c.1177C>T	-	-	1/6 (AFR)	1	BA1, BP4, BP7	17 (8.6%)	3 (25.0%)
p.H397H	c.1191C>T	-	-	1/12682 (FIN)	2	BP4, BP7	2 (1.0%)	-
**SCARB1**								
p.S4S	c.12C>T	-	-	1/10 (AFR)	1	BA1, BP4, BP7	1 (0.5%)	-
p.A7A	c.21G>C	-	-	1/12 (FIN)	2	BP4, BP7	10 (5.1%)	1 (8.3%)
p.G167G	c.501C>T	-	FP	1/2 (EAS)	1	BA1, BP4, BP7	6 (3.0%)	-
p.F301F	c.903C>T	-	-	1/14 (AFR)	1	BA1, BP4, BP7	7 (3.5%)	-
p.A350A	c.1050T>C	-	DFP	1/1 (AFR)	1	BA1, BP4, BP7	156 (78.8%)	9 (75.0%)
-	c.1129-7G>A	-	-	1/8 (AFR)	1	BA1	1 (0.5%)	-
**PLTP**								
p.L303L	c.907C>T	-	-	1/319 (NFE)	2	BP4, BP7	2 (1.0%)	-
p.L323L	c.967T>C	-	-	1/1682 (NFE)	2	BP4, BP7	1 (0.5%)	-
p.G421G	c.1263G>C	-	-	1/100 (ASJ)	2	BP4, BP7	2 (1.0%)	-
p.V448V	c.1344G>C	-	-	1/162 (SAS)	1	BA1, BP4, BP7	2 (1.0%)	-
