# Supplementary material for: Genetic testing in individuals with extreme HDL-C levels: Diagnostic yield and clinical implications from the Tromsø Study
Source: PLoS One. 2026 Apr 20;21(4):e0344627. doi: 10.1371/journal.pone.0344627 (PMC13095017; doi:10.1371/journal.pone.0344627)
Supplement: S4 Table — At the Unit for Cardiac and Cardiovascular Genetics, Oslo University Hospital, we have identified 25 likely causative genetic variants in 41 of 270 individuals which have been genetically tested for abnormal HDL-C levels. The indicated HDL-C levels (mmol/L) of individuals harboring the variants are shown, along with variant pathogenicity classes and the criteria applied according to the ACMG guidelines [21]. HDL-C: HDL-cholesterol. aClass 1: benign; Class 2: likely benign; Class 3: Unknown significance; Class 4: likely pathogenic; Class 5: pathogenic. bCriteria for pathogenicity weighed as very strong (PVS1), strong (PS4, PM3_str, PP4_str), moderate (PS3_mod, PM2–3) or supporting (PS3_sup, PM3–5_sup, PP3). (PDF) [file pone.0344627.s005.pdf]

S4 Table. Genetic variants found at our unit which are likely causes of abnormal HDL-C levels.

		ACMG	
**Variant**	**HDL-C**	**Class[a]**	**Criteria[b]**
**ABCA1**			
p.R282X	< 0.1	5	PVS1, PM2, PM3, PP4_str
p.W590X	< 0.1	4	PVS1, PM2
p.A1046D	< 0.1	5	PS3_sup, PM2, PM3, PP3, PP4_str
p.L1244Q	< 1.0	3	PS3_mod, PM2, PP3
p.R1270X	< 0.1	5	PVS1, PM2, PM3, PP4_str
p.C1429X	< 1.0	4	PVS1, PM2
p.C1477F	< 1.0	4	PS3_sup, PS4, PM2, PM5_sup, PP3
p.Y1532C	< 0.1	5	PS3_sup, PM2, PM3, PP3, PP4_str
p.W1699C	< 0.1	4	PM2, PM3_sup, PP3, PP4_str
p.N1800H	< 0.5	4	PS3_mod, PP3, PP4_str
p.E2106Q	< 0.1	5	PS3_mod, PM2, PM3, PP3, PP4_str
c.302+1G>A	< 0.5	5	PVS1, PS4_sup, PM2
c.814-1G>T	< 0.3	5	PVS1, PM2, PP4_str
c.1758dup	< 0.1	5	PVS1, PM2, PM3, PP4_str
c.2038_2040del	< 0.3	4	PM2, PM4_sup, PP4_str
c.4559+1G>A	< 1.0	4	PVS1, PM2
c.5736del	< 1.0	4	PVS1, PM2
**APOA1**			
p.R184L	< 1.0	4	PS3_sup, PS4, PM2, PP3
c.43+2T>A	< 0.1	5	PVS1, PM2, PP4_str
**CETP**			
p.Q104X	> 3.0	5	PVS1, PM2, PP4_str
p.L290P	> 2.0	3	PS3_mod
p.A291G	> 3.0	3	PS3_mod, PM2
p.E443K	> 3.0	3	PS3_mod, PM2
**LCAT**			
p.R268H	< 0.1	5	PS3_sup, PM2, PM3_str, PP3, PP4_str
p.M276K	< 0.3	5	PS3_sup, PM2, PM3_str, PP3, PP4_str
