# Supplementary figures and images for: Genetic testing in individuals with extreme HDL-C levels: Diagnostic yield and clinical implications from the Tromsø Study
Source: PLoS One. 2026 Apr 20;21(4):e0344627. doi: 10.1371/journal.pone.0344627 (PMC13095017; doi:10.1371/journal.pone.0344627)

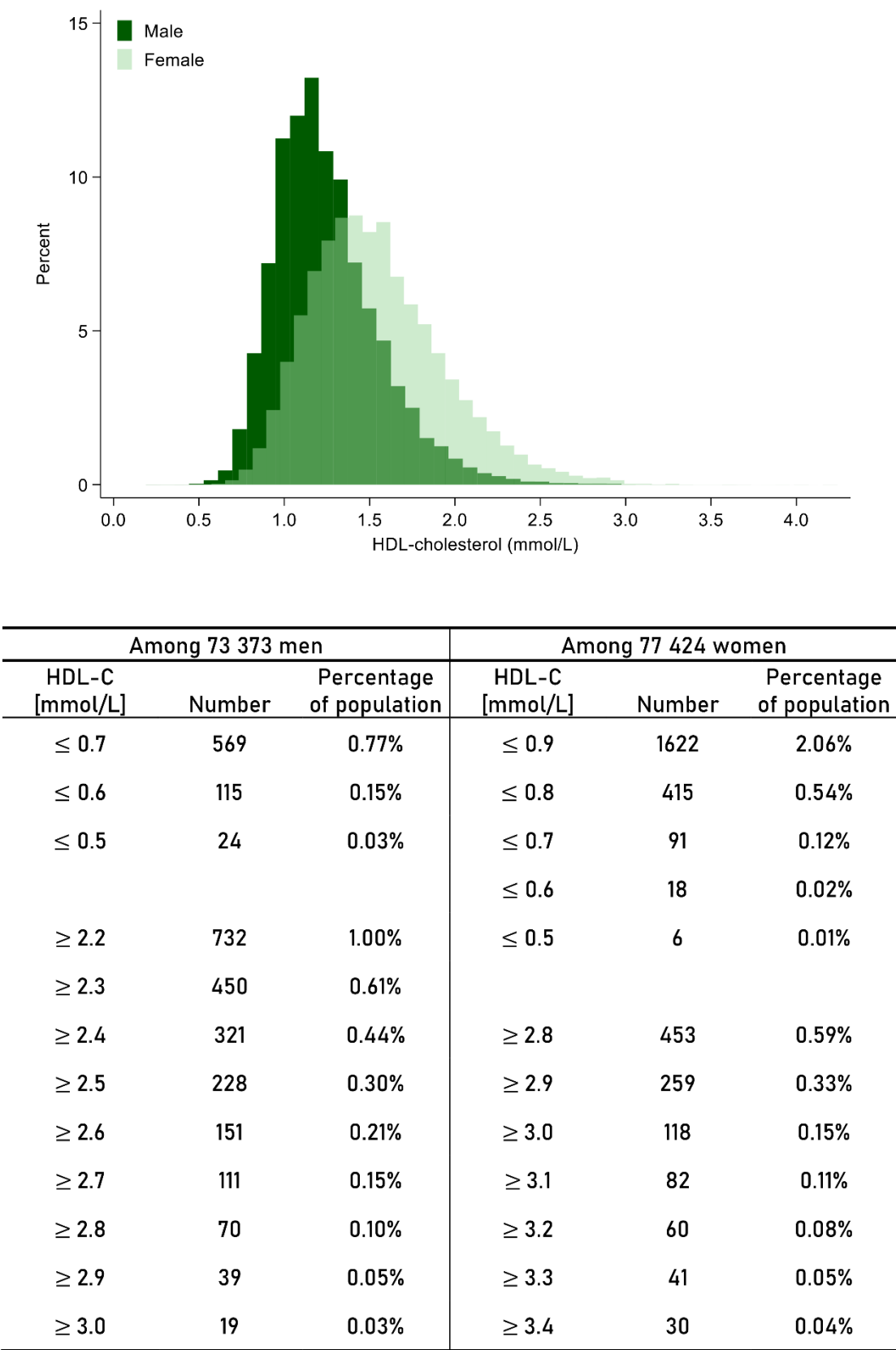

S1 Fig. The distribution of HDL-C.

Supplement: S1 Fig — Two histograms depicting the distribution of HDL-cholesterol (HDL-C) levels in men and women. The data were provided by the Lipid Clinic in Norway, which obtained the information from Fürst Medical Laboratory, Oslo. The dataset pertains to individuals aged 18–49.9 years, primarily measured in general practice, and therefore, the sample is not representative of the general population. A table is included to display the number and percentage of individuals with HDL-C levels above or below specific thresholds. (PDF) [file pone.0344627.s006.pdf]

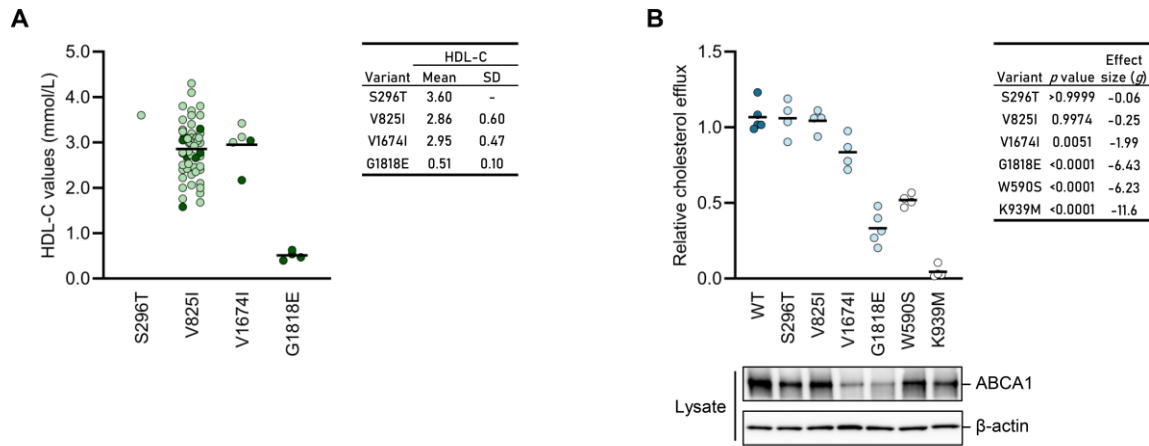

**S2 Fig. Phenotype and functional assay of variants in *ABCA1*.**

Supplement: S2 Fig — Selected ABCA1 missense variants found in the extreme ends of the HDL-C distribution in the Tromsø study were chosen for functional characterization. A) Participant HDL-C levels are given individually (female: light green; male: dark green) and as mean and standard deviation (SD) for each variant. B) Relative cholesterol efflux activity of ABCA1 missense variants (light blue) normalized to wild-type (WT, dark blue) in transiently transfected HEK293 cells. Two loss-of-function controls (white) are shown [36]. p values and effect size (g) compared to WT are given. One representative western blot showing protein amounts in lysates from transiently transfected HEK293 cells is shown. (PDF) [file pone.0344627.s007.pdf]

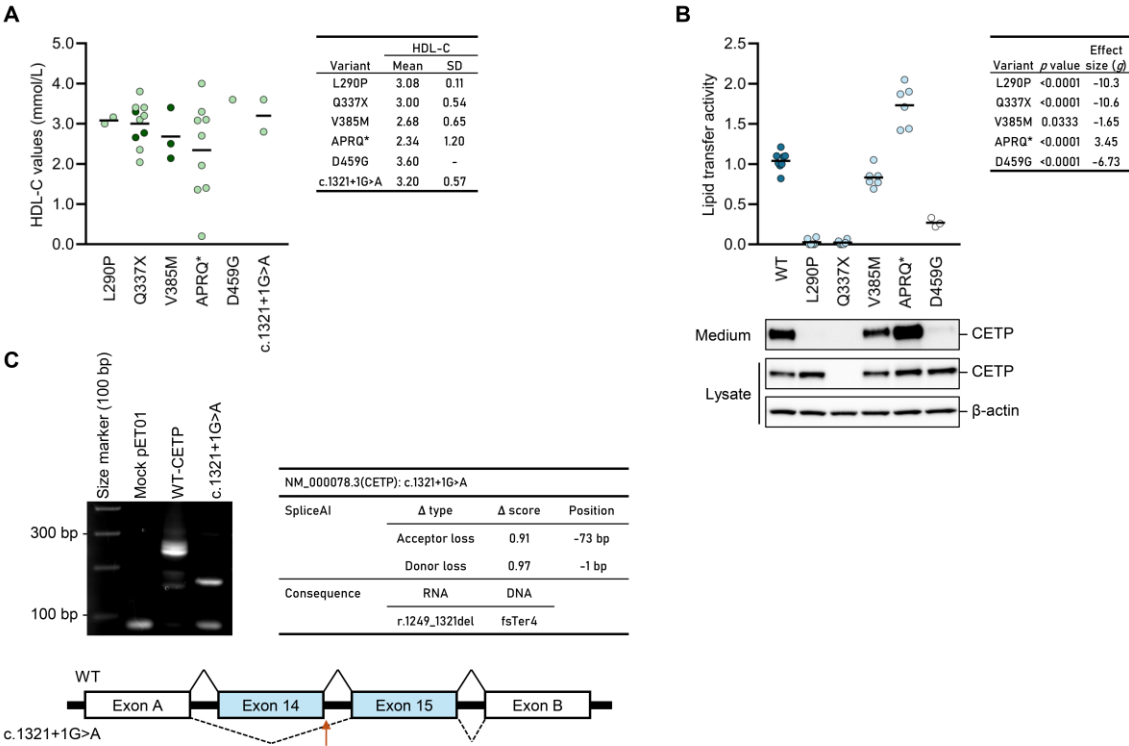

S3 Fig. Phenotype and functional assay of variants in *CETP*.

Supplement: S3 Fig — Selected CETP variants found in the extreme ends of the HDL-C distribution in the Tromsø study were chosen for functional characterization. *APRQ: p.A390P and p.R468Q. A) Participant HDL-C levels are given individually (female: light green; male: dark green) and as mean (SD) for each variant. B) Relative lipid transfer activity of CETP missense variants (light blue) normalized to wild-type (WT, dark blue) in media from transiently transfected HEK293 cells. One loss-of-function control (white) is shown [38]. p values and effect size (g) compared to WT are given. One representative western blot showing protein amounts in media and lysates from transiently transfected HEK293 cells is displayed. C) A DNA fragment spanning intron 13 to intron 15 in CETP was cloned into the pET01 minigene to characterize the consequence of the variant CETP c.1321 + 1G > A. Gel electrophoresis of RT-PCR outcome from transiently transfected HEK293 cells, in silico prediction from SpliceAI and sequencing result (consequence) are shown. Canonical exons are shown as boxes (pET01: white; CETP: blue) separated by introns (black horizontal lines). Solid black lines depict normal splicing (WT), disease-associated splicing indicated by dotted lines. Red arrow indicates the approximate location of the variant. (PDF) [file pone.0344627.s008.pdf]

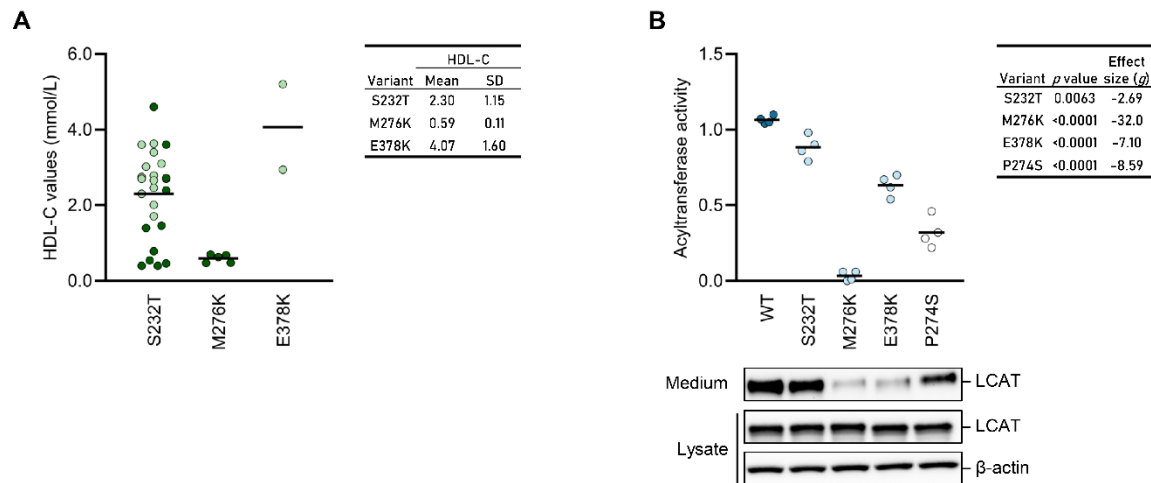

**S4 Fig. Phenotype and functional assay of variants in *LCAT*.**

Supplement: S4 Fig — Selected LCAT missense variants found in the extreme ends of the HDL-C distribution in the Tromsø study were chosen for functional characterization. A) Participant HDL-C levels are given individually (female: light green; male: dark green) and as mean (SD) for each variant. B) Relative acyltransferase activity of LCAT missense variants (light blue) normalized to wild-type (WT, dark blue) in media from transiently transfected HEK293 cells. One loss-of-function control (white) is shown [68]. p values and effect size (g) compared to WT are given. One representative western blot showing protein amounts in media and lysates from transiently transfected HEK293 cells is shown. (PDF) [file pone.0344627.s009.pdf]

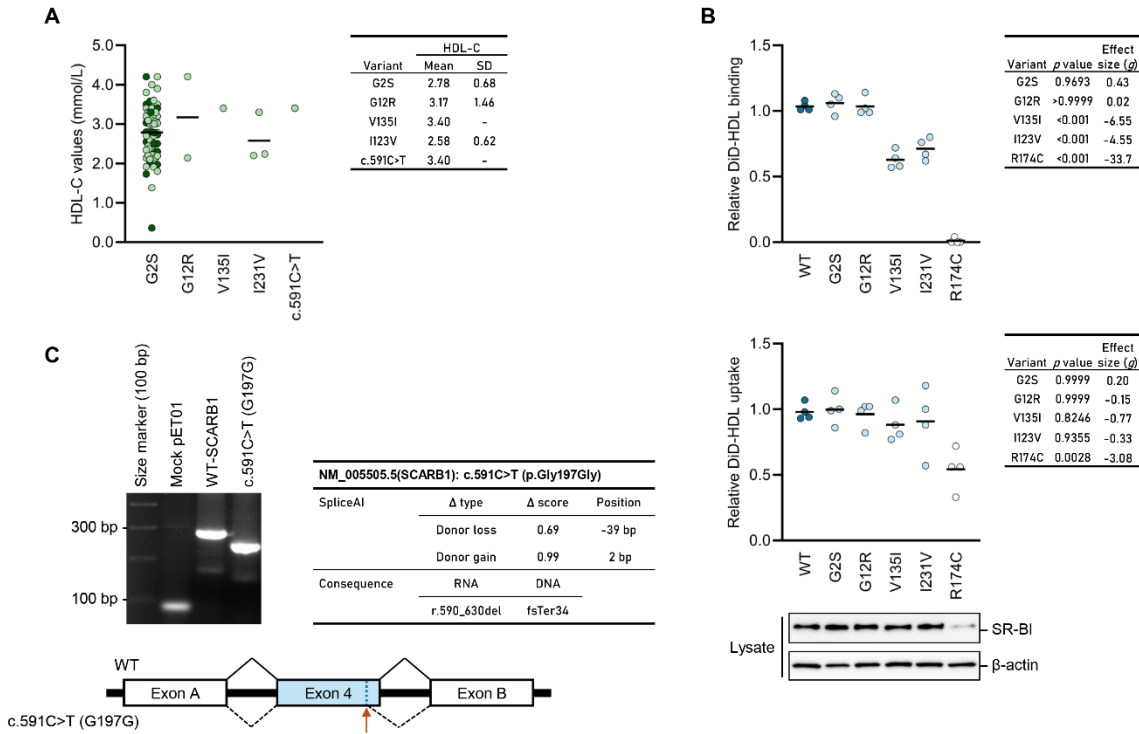

**S5 Fig. Phenotype and functional assay of variants in *SCARB1*.**

Supplement: S5 Fig — Selected SCARB1 variants found in the extreme ends of the HDL-C distribution in the Tromsø study were chosen for functional characterization. A) Participant HDL-C levels are given individually (female: light green; male: dark green) and as mean (SD) for each variant. B) Relative HDL-binding and uptake for SCARB1 missense variants (light blue) normalized to wild-type (WT, dark blue) in transiently transfected HEK293 cells. One loss-of-function control (white) is shown [69]. p values and effect size (g) compared to WT are given. One representative western blot showing protein amounts in media and lysates from transiently transfected HEK293 cells is displayed. C) A DNA fragment spanning intron 3 to intron 4 in SCARB1 was cloned into the pET01 minigene to characterize the consequence of the variant SCARB1 c.591C > T (p.G197G). Gel electrophoresis of RT-PCR outcome from transiently transfected HEK293 cells, in silico prediction from SpliceAI and sequencing result (consequence) are shown. Canonical exons are shown as boxes (pET01: white; SCARB1: blue) separated by introns (black horizontal lines). Solid black lines depict normal splicing (WT), disease-associated splicing indicated by dotted lines. Red arrow indicates the approximate location of the variant. (PDF) [file pone.0344627.s010.pdf]

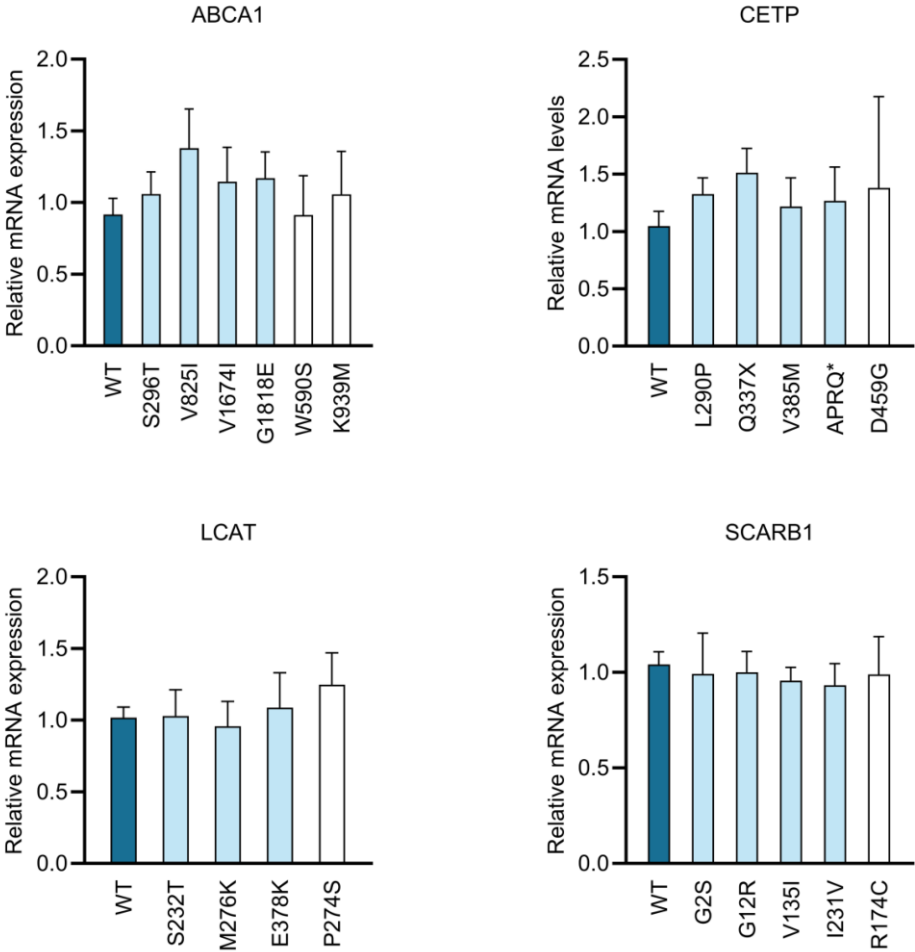

**S6 Fig. mRNA expression.**

Supplement: S6 Fig — RNA was isolated from HEK293 cells transiently transfected with ABCA1, CETP, LCAT or SCARB1 wild-type (WT, dark blue columns), missense variants (light blue columns) and negative control variants (white columns). RNA was transcribed to cDNA, which was analyzed using PrimeTime Predesigned qPCR Assay primers. mRNA amounts were determined and normalized to the housekeeping gene GAPDH by the 2−ΔΔCt method [70] and normalized to WT in three independent experiments. Error bars represent 1 SD. *APRQ: p.A390P and p.R468Q. (PDF) [file pone.0344627.s011.pdf]
