# Supplementary material for: Genetic testing in individuals with extreme HDL-C levels: Diagnostic yield and clinical implications from the Tromsø Study
Source: PLoS One. 2026 Apr 20;21(4):e0344627. doi: 10.1371/journal.pone.0344627 (PMC13095017; doi:10.1371/journal.pone.0344627)

S1 Raw images

S2 Fig raw: ABCA1 in lysates

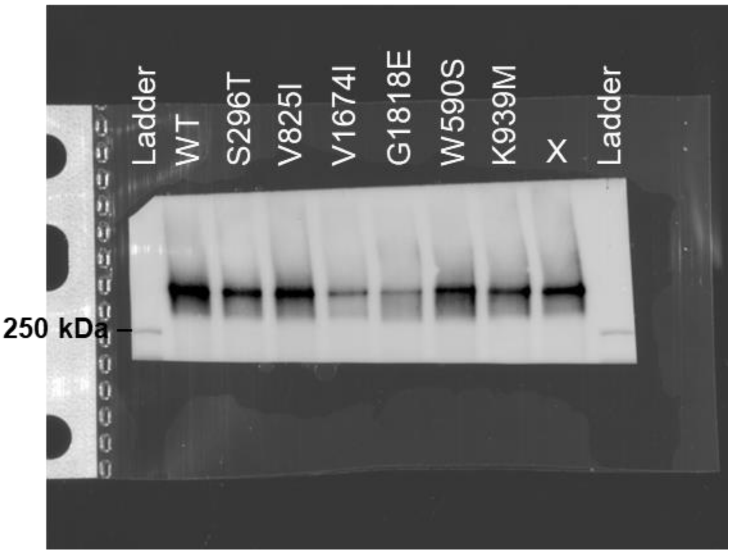

S2 Fig raw: β-actin in lysates

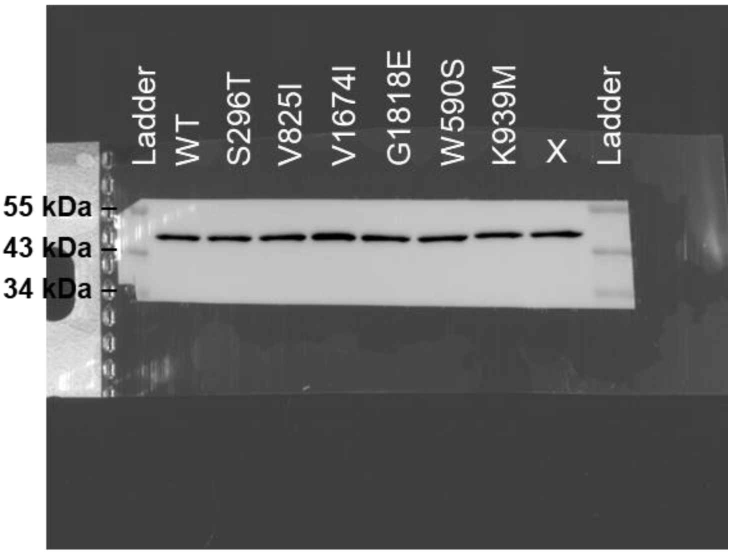

S3 Fig raw: CETP in media

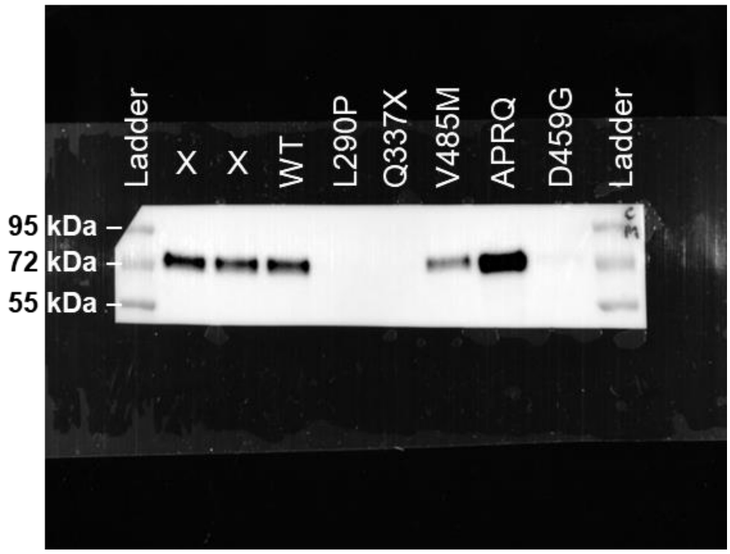

S3 Fig raw: CETP in lysates

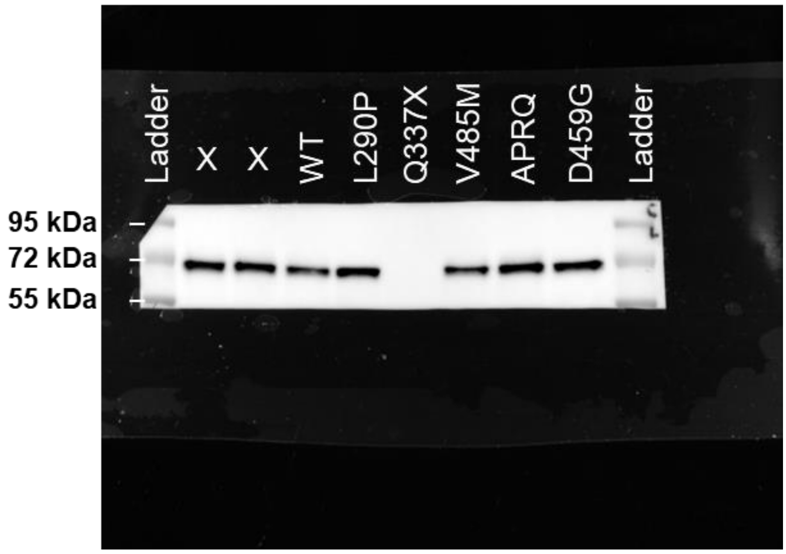

S3 Fig raw: β-actin in lysates

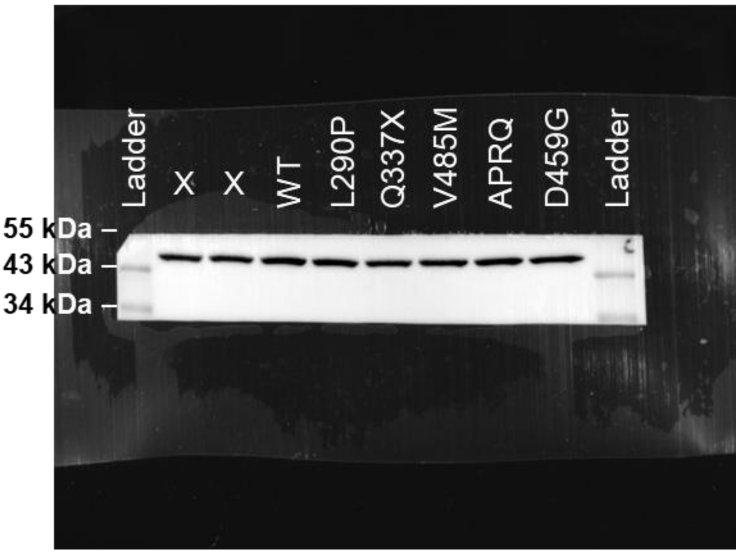

S3 Fig raw: Minigene splice assay for *CETP* c.1321+1G>A

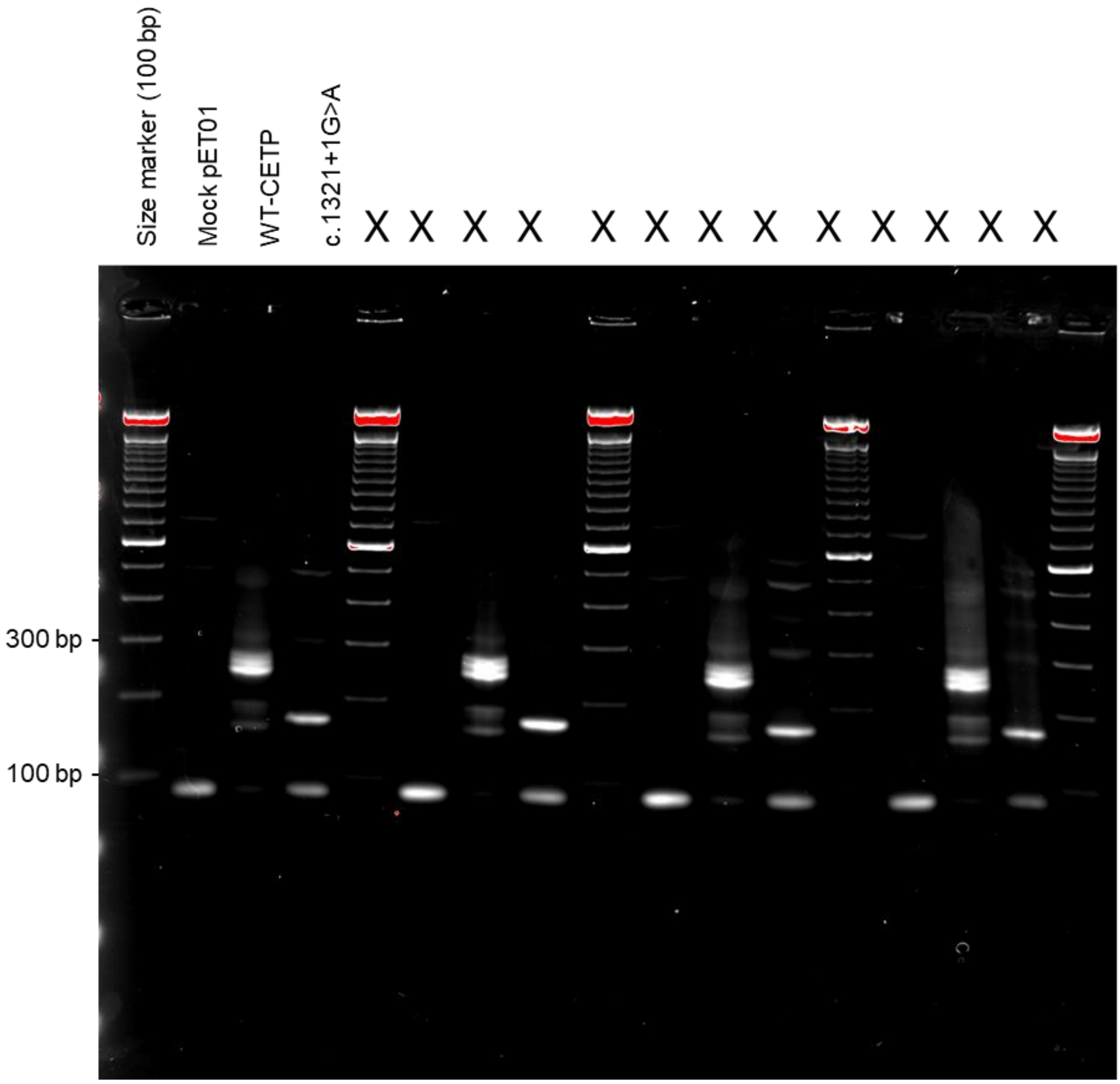

S4 Fig raw: LCAT in media

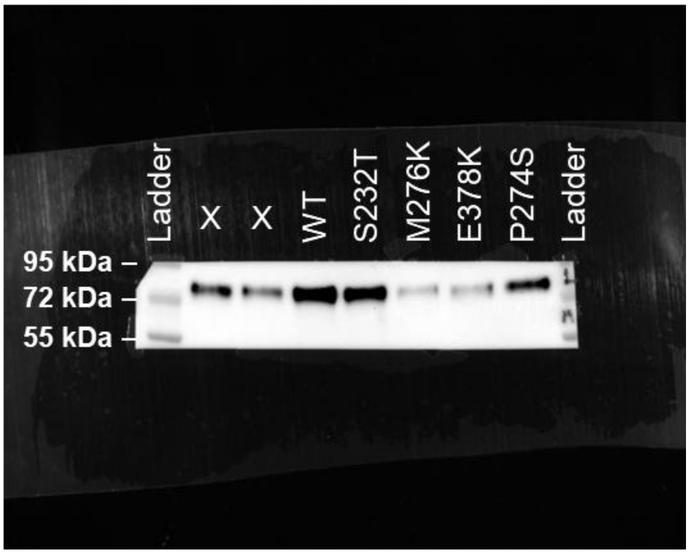

S4 Fig raw: LCAT in lysates

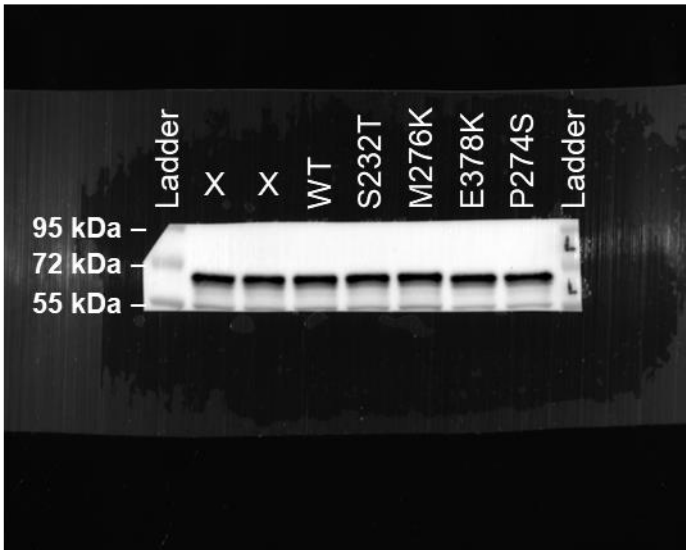

S4 Fig raw: β-actin in lysates

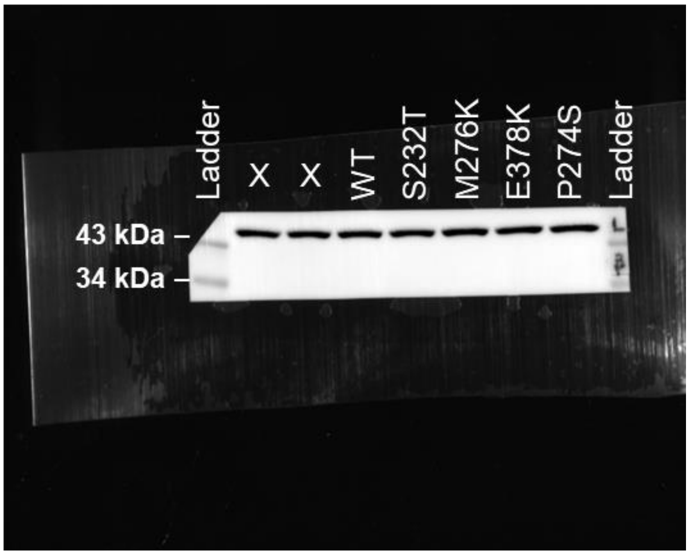

S5 Fig raw: SR-BI in lysates

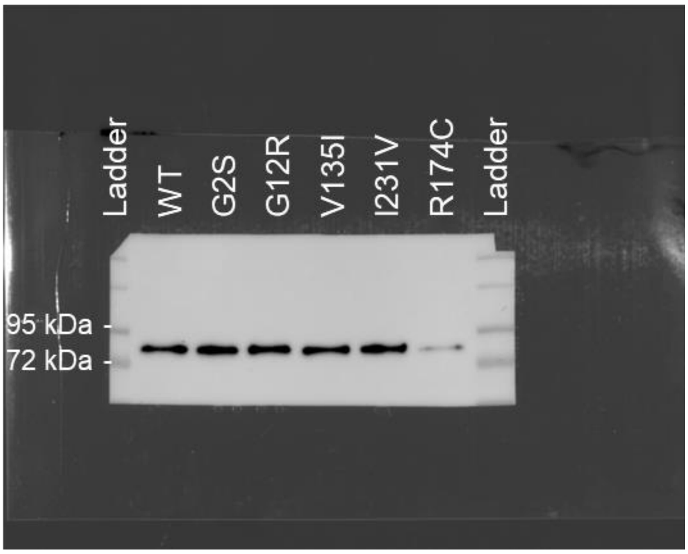

S5 Fig raw: β-actin in lysates

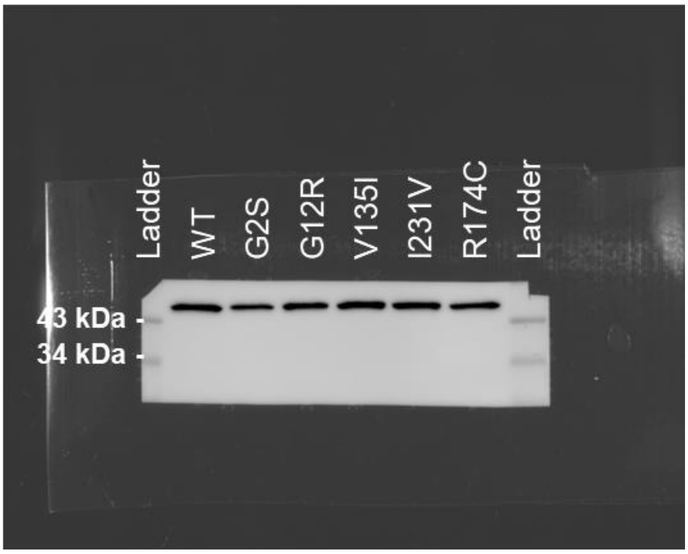

S5 Fig raw: Minigene splice assay for *SCARB1* c.591C>T

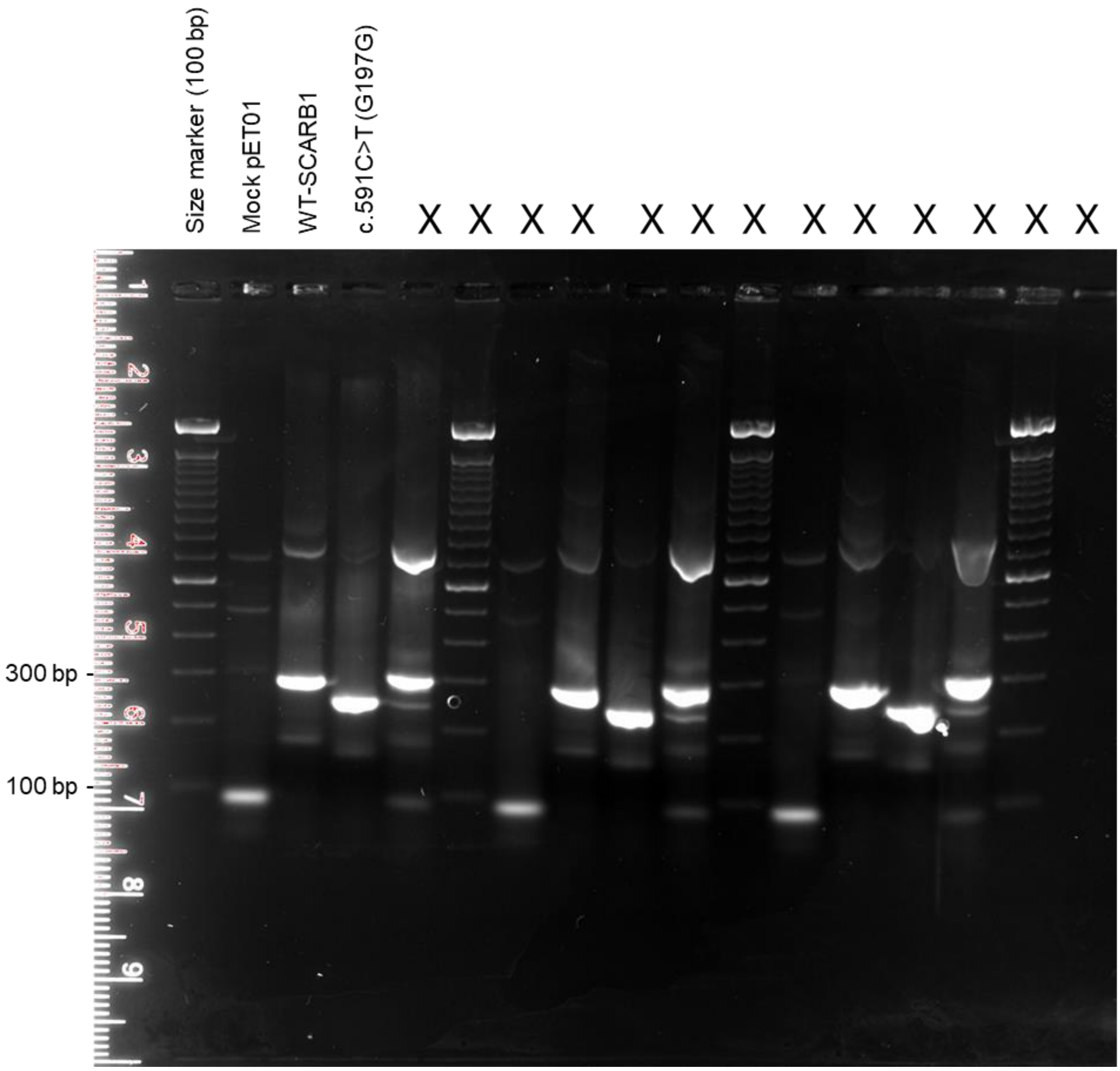

Supplement: S1 Raw Images — (PDF) [file pone.0344627.s012.pdf]
