# Supplementary material for: Genetic testing in individuals with extreme HDL-C levels: Diagnostic yield and clinical implications from the Tromsø Study
Source: PLoS One. 2026 Apr 20;21(4):e0344627. doi: 10.1371/journal.pone.0344627 (PMC13095017; doi:10.1371/journal.pone.0344627)
Supplement: S1 Raw Data — (PDF) [file pone.0344627.s013.pdf]

S1 Raw data

S2 Fig raw: HDL-C values for individuals with variants in the *ABCA1* gene

ABCA1 variant			
**S296T**	**V825I**	**V1674I**	**G1818E**
3.6	2.99	3.12	0.54
	3.10	3.42	0.40
	2.29	3.00	0.63
	2.40	3.04	0.47
	3.50	2.17	
	2.99		
	3.80		
	2.10		
	2.40		
	3.10		
	2.45		
	3.20		
	3.28		
	3.00		
	2.42		
	3.10		
	2.67		
	3.10		
	2.72		
	3.10		
	3.06		
	2.22		
	3.10		
	2.50		
	2.06		
	2.70		
	3.20		
	2.35		
**S296T**	**V825I**	**V1674I**	**G1818E**
	3.10		
	3.80		
	2.80		
	3.60		
	1.68		
	3.40		
	3.04		
	2.53		
	2.00		
	2.77		
	2.66		
	3.30		
	3.24		
	2.92		
	1.76		
	1.88		
	2.00		
	3.70		
	1.58		
	3.05		
	2.47		
	4.30		
	3.60		
	3.10		
	3.08		
	2.76		
	2.78		
	4.10		

S2 Fig raw: Relative cholesterol efflux of ABCA1 variants

WT	S296T	V825I	V1674I	G1818E	W590S	K939M
1.23	1.11	1.06	0.87	0.40	0.57	0.11
1.02	1.04	1.06	0.98	0.48	0.53	0.02
0.99	1.19	0.94	0.78	0.27	0.51	0.03
1.08	0.90	1.11	0.72	0.31	0.47	0.03
1.02				0.20		

S3 Fig raw: HDL-C values for individuals with variants in the *CETP* gene

CETP variant					
**L290P**	**Q337X**	**V385M**	**APRQ**	**D459G**	**c.1321+1G>A**
3.16	2.35	2.14	1.96	3.60	2.80
3.00	3.10	2.50	3.10		3.60
	3.80	3.40	2.70		
	2.77		3.30		
	2.66		4.00		
	3.30		3.08		
	2.04				
	3.20				
	3.40				
	3.40				

S3 Fig raw: Relative lipid transfer activity of CETP variants

WT	L290P	Q337X	V385M	APRQ*	D459G
0.82	0.00	0.00	0.78	1.90	0.22
1.09	0.09	0.00	1.05	1.87	0.33
1.10	0.00	0.04	0.85	1.42	0.26
1.21	0.00	0.00	0.77	2.05	
0.98	0.07	0.07	0.69	1.71	
1.10	0.00	0.02	0.85	1.44	
1.01					
1.00					

S4 Fig raw: HDL-C values for individuals with variants in the *LCAT* gene

LCAT variant		
**S232T**	**M276K**	**E378K**
1.71	0.63	2.94
2.30	0.47	5.20
3.63	0.48	
2.79	0.70	
2.66	0.68	
2.70		
3.02		
1.46		
1.40		
3.60		
3.10		
0.54		

S232T	M276K	E378K
0.4		
0.46		
2.39		
2.72		
4.60		
0.78		
0.4		
2.75		
2.70		
3.40		
2.46		
2.01		
3.60		

S4 Fig raw: Relative LCAT activity of LCAT variants

WT	S232T	M276K	E378K	P274S
1.04	0.90	0.06	0.62	0.28
1.05	0.98	0.00	0.54	0.22
1.10	0.79	0.01	0.67	0.32
1.07	0.86	0.06	0.70	0.46

S5 Fig raw: HDL-C values for individuals with variants in the *SCARB1* gene

HDL-C				
**G2S**	**G12R**	**V135I**	**I231V**	**c.591C>T**
2.29	2.14	3.40	2.24	3.4
2.4	4.20		2.20	
3.5			3.30	
3.06				
2.02				
3.5				
2.5				
2.5				
3.58				
2.78				
2.3				
4.2				
2.55				
2.54				
2.8				
3.4				
0.36				
2.73				
3.03				
1.73				
1.97				
3.3				
2.8				
2.99				
3.8				
2.22				
3.6				
3.08				
2.43				
2.22				
3.1				
2.14				
4.2				
3.4				

G2S	G12R	V135I	I231V	c.591C>T
2.44				
2.6				
3.3				
2.37				
2.7				
3.2				
2.19				
4				
3				
3.28				
3.19				
1.38				
3.1				
2.14				
3.4				
3.9				
2.7				
3.3				
3.1				
2.34				
3.2				
1.92				
3.06				
2.8				
2.2				
1.91				
1.8				
2.1				
3.3				
2.78				
3.09				
3.02				
2.5				
3				

S5 Fig raw: Relative binding of SR-BI variants

WT	G2S	G12R	V135I	I231V	R174C
1.04	1.05	0.99	0.64	0.67	0.00
1.08	1.10	1.02	0.57	0.62	0.00
1.01	0.96	0.99	0.72	0.76	0.04
1.01	1.13	1.14	0.58	0.80	0.00

S5 Fig raw: Selective uptake of SR-BI variants

WT	G2S	G12R	V135I	I231V	R174C
0.98	1.14	0.82	0.81	0.57	0.33
0.93	0.86	1.02	0.88	1.18	0.72
0.94	0.99	1.02	1.07	1.00	0.56
1.07	1.00	0.99	0.77	0.88	0.56

S6 Fig raw: Relative mRNA levels of *ABCA1*, *CETP*, *LCAT*, and *SCARB1*

ABCA1

WT	S296T	V825I	V1674I	G1818E	W590S	K939M
0.89	0.93	1.58	1.42	1.25	1.23	1.24
1.04	1.23	1.07	0.98	0.96	0.77	1.22
0.82	1.02	1.49	1.04	1.30	0.74	0.71

CETP

WT	L290P	Q337X	V385M	APRQ*	D459G
0.94			0.85	0.85	
1.28	1.37	1.33	1.27		
1.02	1.39	1.44	1.40	1.31	
1.02	1.12	1.46	1.35	1.37	
1.23					0.81
0.90					2.51
0.95					0.83
1.01					1.37
1.07	1.43	1.82		1.54	

LCAT

WT	S232T	M276K	E378K	P274S
0.97	1.17	1.12	1.19	
1.05	0.79	0.78	0.76	
1.06	1.17	1.09	1.07	
0.99	0.99	0.84	1.33	
0.96				1.15
0.93				1.11
1.03				1.15
1.16				1.58

SCARB1

WT	G2S	G12R	V135I	I231V	R174C
1.16	1.26	0.90	0.94	0.99	
1.05	0.93	0.91	1.03	1.04	
1.10	0.75	1.09	0.99	0.92	
1.02	1.03	1.10	0.87	0.78	
1.01					0.74
0.98					1.08
0.95					1.20
1.06					0.94
