# Supplementary material for: Genetic testing in individuals with extreme HDL-C levels: Diagnostic yield and clinical implications from the Tromsø Study
Source: PLoS One. 2026 Apr 20;21(4):e0344627. doi: 10.1371/journal.pone.0344627 (PMC13095017; doi:10.1371/journal.pone.0344627)
Supplement: Attachment — Submitted filename: Response to Reviewers.docx [file pone.0344627.s015.docx]

**Response to comments from the reviewers on Manuscript Number: PONE-D-25-62448**

**Response to Reviewer #1**

We thank Reviewer #1 for the thorough and positive evaluation of our manuscript, and for the constructive comments. We are pleased that the reviewer finds the study well conducted, clearly presented, and strengthened by the integration of functional assays and UK Biobank data. Below, we address each comment in detail.

**Minor comments**

**1.** *“In some cases, the term ‘HDL’ is used instead of ‘HDL-C’ to describe HDL concentration. For clarity, consider using ‘HDL-C’ when referring to measured concentrations.”*

**Response:**
We agree and thank the reviewer for pointing this out. The manuscript has been carefully revised to ensure consistent use of **“**HDL-C**”** whenever referring to measured cholesterol concentrations, while “HDL” is reserved for particle/function-related descriptions. These changes have been implemented throughout the text.

**2.** *“On page 4, the authors state that patients with extreme HDL-C levels have been offered genetic testing of five key genes, whereas six genes are analyzed in this study. Please clarify the reason for this difference.”*

**Response:**
Thank you for highlighting this important point. We have now clarified this explicitly in the Introduction.

Briefly, *PLTP* is not part of the routine diagnostic gene panel for HDL-related dyslipidemia at our unit. However, in the context of this study, we had sufficient DNA material to sequence one additional gene beyond the standard panel. We therefore chose to include *PLTP* for two main reasons:

1. We anticipated that PLTP variants could be assessed using the same experimental system as CETP variants (although this later proved not to be feasible for functional assays), and
2. *PLTP* is relatively understudied in the context of extreme HDL-C phenotypes, and we aimed to explore its potential contribution and variant frequency in a population-based setting.

This rationale has now been clearly stated in the revised manuscript (Introduction), to avoid confusion between routine diagnostics and the research-based gene selection in this study.

**3.** *“Please clarify if the reported numbers are based on previously published data or reflect the authors’ own clinical experience.”*

**Response:**
We have now added an explicit statement in the revised manuscript clarifying that these numbers do not represent previously published data, but instead reflect diagnostic activity at our clinical genetic unit.

Specifically, the figures refer to patients referred for and analyzed by genetic testing at the Unit for Cardiac and Cardiovascular Genetics, Oslo University Hospital, over the past 25 years. This additional clarification has been included in the Introduction to ensure transparency regarding the data source.

**4.** *“For consistency, consider replacing the term ‘mutations’ with ‘variants’, and formatting gene symbols according to standard gene nomenclature.”*

**Response:**
We agree with this comment. The manuscript has been revised accordingly:

The term “mutations” has been replaced with “variants” throughout the text, and all gene symbols are now consistently formatted according to standard gene nomenclature.

Once again, we thank Reviewer #1 for the careful reading of our manuscript and for the constructive suggestions, which have helped improve clarity and precision.

**Response to Reviewer #2**

We thank Reviewer #2 for the careful reading of our manuscript and for the positive assessment of the data quality, clinical relevance, and clarity of the presentation. We particularly appreciate the recognition of the clinical importance of raising awareness of the deleterious associations of extreme HDL-C levels with cardiovascular disease. Below, we address each of the reviewer’s concerns in detail.

**Major points**

**1.** *“The definitions of extremely low and high HDL-C levels appear arbitrary and should be supported by relevant references.”*

**Response:**
We thank the reviewer for raising this point. We agree that, without adequate explanation, the HDL-C thresholds used in this study (≤ 0.5 mmol/L and ≥ 3.0 mmol/L) may indeed appear arbitrary, and we acknowledge that this was not sufficiently clear in the original version of the manuscript.

These thresholds were not derived from population-based percentiles, but were historically defined by clinicians at our diagnostic unit more than a decade ago, within a markedly different clinical and scientific landscape. At that time, genetic testing was resource-intensive and costly, laboratory capacity was limited, and clinical awareness of HDL-related disorders was low. Healthcare systems, including the Norwegian public healthcare system, emphasized cost–benefit considerations, avoidance of unnecessary testing, and prioritization of limited genetic resources toward conditions with established therapeutic consequences. Together, these factors favored the use of particularly strict eligibility criteria for genetic testing.

Importantly, these stringent thresholds were selected in clinical settings to enrich for individuals with a high likelihood of monogenic causes of extreme HDL-C levels. We have now added references supporting the biological relevance of the applied cutoffs and reflecting the evidence base that informed the original diagnostic criteria.

Since then, the clinical and scientific landscape has changed substantially. There is now increased recognition of the association between extreme HDL-C levels and adverse outcomes, greater interest in HDL biology, and markedly reduced costs and increased capacity for genetic testing.

We fully agree with the reviewer that these historical thresholds were unusually strict. Importantly, recognizing this limitation is a central motivation for the present study. By leveraging population-based data, we demonstrate that the previous cutoffs introduced a pronounced sex bias and captured only a small fraction of individuals with potentially pathogenic variants. In response, and partly informed by the work conducted during the preparation of this manuscript, the criteria for clinical genetic testing at our unit have now been revised toward less extreme, sex-specific thresholds. The Methods and Discussion sections have been revised to more clearly explain the origin of the original cutoffs, their limitations, the clinical reasoning underlying the updated thresholds, and the references used.

**2.** *“The description of the supplementary data (Figures S1 to S6) in the text of the manuscript is unsatisfactory. These data should be presented in more detail. Figure S6 should be described in the Results section, not in the Discussion.”*

**Response:**

The supplementary figures are now described in greater detail in the Results section, with explicit references to the relevant figures. And to improve logical flow and clarity, the supplementary figures have been renumbered: the former S6 Fig is now S1 Fig, and the previous S1–S5 Figs are now S2–S6 Figs.

**Minor points**

**1.** *“The terms ‘HDL levels’ and ‘HDL-C levels’ seem to be used as synonyms.”*

**Response:**
We agree with this comment. The manuscript has been revised to ensure consistent and appropriate use of terminology: “HDL-C” is now used when referring to measured cholesterol concentrations, whereas “HDL” is reserved for particle- or function-related contexts.

**2.** *“Table S4 contains important data on the pathogenicity of genetic variants and should be moved to the main manuscript.”*

**Response:**
We appreciate the reviewer’s suggestion and understand the rationale for highlighting these data. Table S4 summarizes genetic variants previously identified at our diagnostic unit and is intended to provide additional clinical context based on our cumulative experience with HDL-related disorders. These variants are independent of the Tromsø Study cohort and do not originate from the population-based material analyzed in the present study.

For this reason, we chose to place Table S4 in the supplementary material, in order to clearly distinguish historical diagnostic findings from the primary results derived from the Tromsø Study. We felt that including these data in the main manuscript might risk blurring this distinction.

That said, we acknowledge the reviewer’s point that the information may be of interest to readers. If the reviewer or editor considers it important to move Table S4 to the main manuscript, we would of course be happy to do so or to revise its presentation accordingly.

**3.** *“LPL directly contributes to the circulating HDL pool by producing surface remnants of triglyceride-rich lipoproteins rich in phospholipids and free cholesterol. Please revise.”*

**Response:**
Thank you for this clarification. When shortening the Discussion section (Minor comment #4), we decided to remove the description of the roles of each protein mentioned in “Limitations of the study” (ANGPTL3, LPL, LAL, hepatic and endothelial lipase). If the reviewer or editor finds it important to include these descriptions, we will include them in the manuscript and make sure that LPL’s role is correctly explained.

**4.** *“The Discussion section is too lengthy and should be shortened.”*

**Response:**
We agree and have substantially revised the Discussion section. The total length has been reduced from 2264 words to 1823 words, corresponding to an approximate 20% reduction, while preserving the key clinical, methodological, and interpretative points. Redundant passages have been removed or condensed, and the overall structure has been tightened to improve readability.

Once again, we thank Reviewer #2 for the thoughtful and constructive feedback, which has led to improvements in clarity, structure, and contextualization of the manuscript.

**Response to Academic Editor**

We thank the Academic Editor for the careful assessment of our manuscript and for the clear and constructive guidance, which has helped us improve the manuscript’s compliance with *PLOS ONE*’s policies and standards.

**1.** *Please ensure that your manuscript meets PLOS ONE's style requirements, including those for file naming.*

**Response:**

We have carefully reviewed the manuscript and all associated files and ensured that they comply with *PLOS ONE*’s style and file-naming requirements.

**2.** *We note that the grant information you provided in the ‘Funding Information’ and ‘Financial Disclosure’ sections do not match.*

**Response:**

All funding-related text has been removed from the manuscript. The amended Funding Statement has been included in the revised cover letter, as requested.

**3.** *Please remove any funding-related text from the manuscript and let us know how you would like to update your Funding Statement. Currently, your Funding Statement reads as follows:*

*“This research was supported by the Norwegian Health Association (https://nasjonalforeningen.no/) (Grant No. 22741 received by K.B.). The funding body had no role in study design, data collection and analysis, decision to publish, or preparation of the manuscript.”*

*Please include your amended statements within your cover letter; we will change the online submission form on your behalf.*

**Response:**

The amended statement has been included in the revised cover letter.

**4.** *We note that there is identifying data in the Supporting Information file “S1_Table.pdf” Due to the inclusion of these potentially identifying data, we have removed this file from your file inventory. Prior to sharing human research participant data, authors should consult with an ethics committee to ensure data are shared in accordance with participant consent and all applicable local laws.*

*Data sharing should never compromise participant privacy. It is therefore not appropriate to publicly share personally identifiable data on human research participants.*

*Please remove or anonymize all personal information (<specific identifying information in file to be removed>), ensure that the data shared are in accordance with participant consent, and re-upload a fully anonymized data set. Please note that spreadsheet columns with personal information must be removed and not hidden as all hidden columns will appear in the published file.*

**Response:**

We have revised S1 Table to further anonymize the data by replacing specific participant ages with age groups and have ensured that the data shared are in accordance with participant consent and applicable regulations. The updated, anonymized S1 Table has been re-uploaded to the file inventory. Corresponding changes have also been made in Table 2 in the main manuscript.

**5.** *We note that you have included the phrase “data not shown” in your manuscript. Unfortunately, this does not meet our data sharing requirements. PLOS does not permit references to inaccessible data. We require that authors provide all relevant data within the paper, Supporting Information files, or in an acceptable, public repository. Please add a citation to support this phrase or upload the data that corresponds with these findings to a stable repository (such as Figshare or Dryad) and provide and URLs, DOIs, or accession numbers that may be used to access these data. Or, if the data are not a core part of the research being presented in your study, we ask that you remove the phrase that refers to these data.*

**Response:**

We have removed the phrase “data not shown” and the corresponding statement from the figure legend of S3 Fig, as the information was not essential to the presentation or interpretation of the results.

**6.** *Please include your full ethics statement in the ‘Methods’ section of your manuscript file. In your statement, please include the full name of the IRB or ethics committee who approved or waived your study, as well as whether or not you obtained informed written or verbal consent. If consent was waived for your study, please include this information in your statement as well.*

**Response:**

The ethics statement has been revised in the Methods section to explicitly state that written informed consent was obtained from all participants.

**7.** *If the reviewer comments include a recommendation to cite specific previously published works, please review and evaluate these publications to determine whether they are relevant and should be cited. There is no requirement to cite these works unless the editor has indicated otherwise.*

**Response:**

No specific recommendations to cite additional publications were made by the reviewers.

**8.** *Please review your reference list to ensure that it is complete and correct. If you have cited papers that have been retracted, please include the rationale for doing so in the manuscript text, or remove these references and replace them with relevant current references. Any changes to the reference list should be mentioned in the rebuttal letter that accompanies your revised manuscript. If you need to cite a retracted article, indicate the article’s retracted status in the References list and also include a citation and full reference for the retraction notice.*

**Response:**

The reference list has been carefully reviewed. No retracted articles are cited.

**Additional changes:**

In addition to the revisions described above, we have carefully reviewed and refined the pathogenicity interpretation of several genetic variants based on constructive feedback received from a member of a PhD defense committee, who had access to the manuscript through its inclusion in a doctoral dissertation. Corresponding updates have been made to Tables S2–S4. These refinements enhance the scientific rigor and clarity of the manuscript and do not alter the main findings or conclusions of the study.
